# Dietary and non-dietary risk factors associated with excess body mass and abdominal obesity in adolescents from rural regions of southern Poland: a cross-sectional study

**DOI:** 10.3389/fpubh.2025.1578906

**Published:** 2025-06-18

**Authors:** Ewa Błaszczyk-Bębenek, Przemysław Holko, Paweł Kawalec, Beata Piórecka

**Affiliations:** Department of Nutrition and Drug Research, Institute of Public Health, Faculty of Health Sciences of the Jagiellonian University Medical College, Kraków, Poland

**Keywords:** abdominal obesity, adolescents, excess body mass, overweight, obesity, risk factors

## Abstract

Excess body mass, including abdominal obesity, has multifactorial causes that are closely linked to various aspects of child development. The prevalence of excess body mass in the pediatric population in Poland is increasing. This study aimed to assess risk factors for excess body mass, including abdominal obesity, among adolescents from southern Poland. A total of 381 adolescents were included to determine the prevalence of overweight and obesity based on body mass index (BMI ≥ 85th percentile) and of abdominal obesity based on waist-to-height ratio (WHtR ≥0.5). Lifestyle habits, along with dietary and sociodemographic data, were assessed as independent risk factors for excess body mass and abdominal obesity using regression analysis. Excess body mass and abdominal obesity were reported in 17 and 8.1% of participants and were positively correlated with body dissatisfaction, fear of becoming obese, physical exercise after eating to prevent weight gain, following a weight-loss diet, receiving comments about one’s appearance, accepting one’s figure but starting a diet due to environmental or media pressure, and more frequent snacking. Excess body mass was positively correlated with low physical activity at school, while abdominal obesity was linked to a less health-promoting diet. Our study indicates the presence of several modifiable risk factors associated with excess body mass and abdominal obesity. These findings may help guide future multifactorial prevention strategies aimed at reducing obesity and its long-term consequences in adulthood.

## 1 Introduction

Excess body mass, including overweight and obesity, is a multifactorial condition with genetic, psychosocial, and environmental causes (1–3). It represents a major health problem and a public health challenge (1). The prevalence of overweight and obesity among children and adolescents is increasing at an alarming rate and is influenced by familial, environmental, socioeconomic, and cultural factors (4–7). The economic transition in Poland has led to improved living conditions, which in turn have influenced lifestyle changes in families, which differ from patterns observed in Western Europe (8). The link between excess body mass and adverse health outcomes, such as elevated risk of type 2 diabetes, cardiovascular disease, metabolic disorders, reduced quality of life, and shorter life expectancy, is well documented (6, 9, 10).

In addition to excess body mass (general obesity), abdominal fat accumulation further increases the risk of cardiometabolic conditions, such as dyslipidemia, hypertension, hyperglycemia, insulin resistance, and type 2 diabetes, in the pediatric population (11–13). Moreover, abdominal obesity in children and adolescents was associated with respiratory disorders, including asthma, and with progression to adult obesity (14, 15). Therefore, early intervention strategies are essential to mitigate these risks.

During the school years, inappropriate dietary patterns may be adopted from peers. Poor eating habits, such as irregular meal consumption, skipping breakfast, and frequent intake of high-calorie, low-nutrient foods, are among the factors associated with excess body mass (4, 16, 17). Addressing abdominal obesity in adolescents, similar to managing excess body mass, requires a multifaceted approach due to the involvement of various contributing factors. Dietary shifts during adolescence, particularly toward carbohydrate-rich diets, are associated with visceral fat accumulation. Additionally, reduced physical activity, prolonged screen time, and short sleep duration – characteristic of this life stage – further increase the risk of obesity, including abdominal obesity (18, 19).

The increasing incidence of excess body mass represents a global public health challenge. Available studies conducted in different populations of children and adolescents reported inconsistent results, as there are regional differences in the prevalence and trends of obesity (3, 4, 6). Many of the strategies aimed at preventing and treating excess body mass point to a strong need for locally focused measures. Understanding the regional prevalence of overweight and obesity is critical to developing effective interventions and local policies to improve young people’s health and to reduce inequalities caused by modifiable risk factors (3, 4, 6, 20, 21). In a study conducted between 1995 and 2006, Jodkowska et al. reported overweight and obesity rates among adolescents aged 13–15 years from five voivodships in Poland. While there were some regional differences, they were not related to rural versus urban residence. In addition, no differences were found in the rates of physical activity and sedentary lifestyle or in the eating habits across regions. No significant association was found between urbanization and the prevalence of excess body mass; however, the authors anticipated a growing trend of increasing rates in rural areas (21).

Based on a review of the literature, we identified factors – particularly modifiable ones – associated with excess body mass, including abdominal obesity, that may have implications for public health strategies. To better understand the relationship between excess body mass and various influencing factors, we examined variables related to dietary habits (e.g., frequency of consumption and general indicators of diet quality), lifestyle behaviors, and attitudes toward overweight, obesity, and self-perceived body mass. Specifically, we assessed selected sociodemographic, nutritional, health-related, and psychosocial factors associated with the occurrence of excess body mass (body mass index [BMI] ≥ 85th percentile) (22, 23) and abdominal obesity (waist-to-height ratio [WHtR] ≥ 0.5) (24) in adolescents from rural regions of the Małopolska Voivodeship in southern Poland.

## 2 Materials and methods

### 2.1 Data collection

This cross-sectional study was conducted between 2016 and 2019, mostly in the rural areas of the Małopolska Voivodeship in Poland. The nonrandom selection of participants with convenience sampling was used. The invitation to participate in the study was sent to public schools on the basis of the Register of Schools and Educational Institutions and meetings with school representatives at the sanitary inspection stations of the voivodeship. The survey was conducted in 9 schools whose principals agreed to take part in the project. Participants aged 6 to 17 years, of both sexes, who had obtained consent from their legal guardians were included in the study. For participants aged 16 years or older, personal consent was also required. Eligible participants had to be able to complete the questionnaire independently and undergo anthropometric measurements under the study conditions. Participants were excluded if they were outside the specified age range, failed to provide the necessary consent, were absent on the day of the study, were unable to complete the questionnaire independently, or could not undergo anthropometric measurements. The initial sample included 475 respondents aged between 7 and 17 years. Seven records from participants aged 7 to 9 years were excluded from the analysis. In addition, 87 cases with incomplete data were excluded from the study.

### 2.2 Anthropometric parameters

Anthropometric measurements of participants included body mass (kg), height, and waist and hip circumferences. Blood pressure was also measured. Height was measured using a MARSDEN-HM-250P stadiometer (Rotherham, United Kingdom). The TANITA TBF-300 (Tokyo, Japan) MA scale was used to measure body mass and analyze the body composition. BMI was calculated for each participant, taking into account sex and age, and was used to assess the nutritional status. BMI was interpreted using centile grids from the Polish OLAF study (22). Overweight was defined as a BMI between the 85th and 95th percentile and obesity as a BMI above the 95th percentile (22, 23). For body fat percentage, reference data from a study by McCarthy et al. were used (25). The incidence of abdominal obesity was assessed on the basis of waist circumference (WC), waist-to-hip ratio (WHR), and WHtR. WC was measured at the midpoint between the lowest rib and the iliac crest and expressed in centimeters. Abdominal obesity was classified using the 90th percentile threshold, based on reference grids for children and adolescents in Poland (24). The WHR was calculated using the following empirical formula: WC (cm)/hip circumference (cm), and WHtR as: WC (cm)/height (cm). Adult cut-off values for WHR were 0.90 for boys and 0.85 for girls (24). For WHtR, a cut-off point of 0.5 was applied for both sexes (24). WHtR scores were included in the model to assess the risk of abdominal obesity, as it enables rapid and early identification of abdominal obesity and its cut-off has been validated in multiple countries.

Blood pressure (BP) was measured in accordance with established guidelines for children and adolescents (26), using an automatic oscillometric monitor (Bosch – Boso Karat Professional, Jungingen, Germany) with an age-appropriate cuff. Systolic BP (SBP) and diastolic BP (DBP) were evaluated against current reference values from the OLAF study (27) and classified according to the 2019 guidelines of the Polish Society of Hypertension (28). In younger children, BP values above the 95th centile were used as the threshold for diagnosing hypertension. For adolescents aged 16 years or older, a fixed cut-off value of ≥ 140/90 mmHg was applied (28).

### 2.3 The questionnaire

The anonymous survey questionnaire consisted of 5 parts and included questions on diet, lifestyle, and sociodemographic characteristics. It was developed by the Committee of Human Nutrition Polish Academy of Sciences (29). The first part of the questionnaire (part A) contained questions on dietary habits in the past year (i.e., number of meals per day, time of eating, snacking between meals, and foods consumed as snacks between meals). Part B concerned the frequency of consuming selected foods and beverages in the past year. Respondents indicated the frequency of consumption on a six-point scale where 1 indicated “never,” 2 – “1 to 3 times a month,” 3 – “once a week,” 4 – “few times per week,” 5 –“once a day,” and 6 – “few times per day.” The Diet-Quality-Index (DQI) indicator from KomPAN was used for a comprehensive assessment of diet quality. The index covers 22 food groups, including 10 groups with potentially beneficial health effects and 12 groups with potentially adverse health effects. The indices were calculated according to the KomPAN procedure by summing the frequency of consumption (times/day) of the 22 food groups. The interpretation of the DQI is based on the assumption that higher scores reflect a greater intensity of health-promoting nutritional characteristics and overall better diet quality (29). In part C, participants were asked about their attitudes toward their own body mass and diet. Based on the Storz and Greene’s silhouette test, which was validated in a previous study (30), they chose a silhouette that was most similar to their own perceived and ideal silhouette. In part D, respondents were asked about selected lifestyle characteristics, such as dietary habits and consumption of out-of-home meals. Next, respondents were asked if they had ever smoked cigarettes or consumed alcohol. Additional questions concerned sleep duration, separately for weekdays and weekend days (three response options: “6 or less hours/day,” “7 or 8 h/day,” and “9 or more hours/day”). The lifestyle section included questions on the amount of time spent daily watching television or using a computer. This was followed by a self-assessment of physical activity at home and during free time, categorized into three intensity levels: low, moderate, and high. Finally, part E covered sociodemographic data, including self-reported financial – specifically, whether parents had sufficient funds to buy desired food – as well as information on parental occupational status.

### 2.4 Statistical analysis

Based on age categories of BMI, participants were divided into the following groups: underweight and normal weight, overweight and obesity (excess body mass), and with or without abdominal obesity. The results were presented as mean with standard deviation and median with interquartile range for continuous variables and as frequencies for categorical variables. The Agresti-Coull approach was used to calculate the 95% confidence interval (CI) for binomial proportions (i.e., 95% CI for the proportion of BMI categories) (31). The χ^2^ Pearson test for categorical variables and the Wilcoxon rank-sum test for continuous variables were used for an unadjusted comparison between study groups divided by sex (female vs. male) or excess body mass status (overweight or obesity by BMI vs. other BMI categories). Agreement between the definitions of excess body mass was assessed by percentage agreement and the Cohen’s *κ* coefficient. The Wilcoxon rank-sum test for nutritional indices was used for unadjusted comparison between participants with excess body mass and the remaining participants. Generalized linear models (binomial distribution with logit link function) with robust variance estimators were used to analyze the difference in outcome between subgroups (controlling for other variables that might affect the outcome) and to identify the variables most strongly associated with an outcome (being overweight or obese or having abdominal obesity). Variables were selected using a backward elimination method (threshold *p*-value of 0.2). Interactions between variables were included if their inclusion substantially improved the fit of the model to the data (i.e., >10% increase in log-likelihood or pseudo-R2). The selection and assessment of the models were based on the Box-Cox test, modified Park test, and log-likelihood. Predictive margins were presented as adjusted probabilities of being overweight or obese. Average marginal effects (for continuous variables) or contrasts of predictive margins (for categorical variables) were presented as adjusted differences in the probability of being overweight or obese or having abdominal obesity, with standard errors calculated using the delta method.

The study included all participants who completed the questionnaire. Missing data were excluded from the analysis of specific outcomes when they had no or minimal impact on the results (e.g., single missing values: waist circumference, 1 case; weight, 1 case; body composition, 2 cases; number of siblings, 1 case). No multiplicity correction was implemented. A *p*-value of less than 0.05 was considered significant. Data were prepared and analyzed using StataNow/SE 18.5 (StataCorp., College Station, TX, USA) and OriginPro 2024b (OriginLab Corp., Northampton, MA, USA). The study was carried out according to the Strengthening the Reporting of Observational Studies in Epidemiology Statement (32).

## 3 Results

### 3.1 Description of the study group

The study included 381 children and adolescents (boys, 44.9%; girls, 55.1%) mean aged 13.1 ± 1.8 years, of whom 81.9% lived in rural areas. Girls (55.7%) attended secondary school significantly more often than boys, who mostly attended primary school (56.1%; *p* < 0.05). In most cases, both parents worked professionally (48.3%) and always had enough money to buy the food they wanted (85.3%). The baseline sociodemographic characteristics of the study group are presented in Table 1.

**Table 1:** Baseline sociodemographic characteristics of the study group.

Variable		Total *n* = 381	Boys *n* = 171	Girls *n* = 210	*p*
Age	Mean±SD Median (IQR)	13.12 ± 1.80 13.12 (11.57–14.69)	13.00 ± 1.81 12.77 (11.56–14.58)	13.22 ± 1.78 13.34 (11.64–14.74)	0.460
		%	%	%	
Place of living	Village	81.9	79.5	83.8	0.203
	Town	2.6	1.8	3.3	
	City	15.5	18.7	12.9	
Type of school	Primary	49.6	56.1	44.3	**0.021**
	Secondary	50.4	43.9	55.7	
Having sibling(s)	Yes	91.1	90.6	91.4	0.789
Parental occupational status	Neither parent working	9.7	9.9	9.5	0.221
	Only mother working	10.8	9.4	11.9	
	Only father working	31.2	26.9	34.8	
	Both parents working	48.3	53.8	43.8	
Having enough money to buy food	Never	4.2	4.1	4.3	0.969
	Sometimes	4.7	4.7	4.8	
	Most of the time	5.8	6.4	5.2	
	Always	85.3	84.8	87.7	

IQR, interquartile range; *n*, number; SD, standard deviation. Statistically significant values are in bold, with the significance level set at *p* < 0.05. Data are presented as % of participants unless otherwise indicated. The χ^2^ Pearson test (categorical variables) or Wilcoxon rank-sum test (continuous variable) were applied.

The overall prevalence of overweight and obesity in children and adolescents was 16.8%, and there were no significant differences between boys and girls (Table 2). Compared with the general Polish population, children with obesity and underweight based on BMI were slightly underrepresented (obesity, 3.7%; Agresti-Coull 95% CI: 2.1 to 6.1% vs. 5% in the general population; underweight, 3.4%; Agresti-Coull 95% CI: 1.9 to 5.8% vs. 10% in the general population). On the other hand, children with overweight were overrepresented (overweight, 13.1%; Agresti-Coull 95% CI: 10.1 to 16.9% vs. 10% in the general population). The prevalence of abdominal obesity for all study participants was 11.1% based on WC, 6.0% based on WHR, and 8.1% based on WHtR. Based on WC and WHtR, abdominal obesity was significantly more common in boys (11.7 and 10.5%, respectively) than in girls (10.5 and 6.2%, respectively). Conversely, the WHR indicated a significantly higher prevalence of abdominal obesity in girls than in boys (6.2% vs. 5.8%, respectively; *p* < 0.001). Most participants had normal SBP and DBP values, but increased SBP values were significantly more common in boys than in girls (*p* = 0.003).

**Table 2:** Nutritional and health status of the study group.

Variable	Measure	Total *N* = 381	Sex			Overweight /Obesity by BMI		
			Boys *n* = 171	Girls *n* = 210	*p*	No *n* = 317	Yes *n* = 64	*p*
Weight, kg	Mean±SD Median (IQR)	49.11 ± 13.80 48.23 (38.89–56.20)	50.52 ± 15.54 49.44 (37.6–58.32)	48.00 ± 12.10 47.43 (39.64–55.63)	0.531	45.62 ± 10.50 45.90 (37.24–52.90)	66.31 ± 15.34 64.10 (55.50–76.91)	0.124
Height, cm	Mean±SD Median (IQR)	157.89 ± 11.89 158.00 (149.50–165.50)	158.90 ± 13.34 159.00 (148.00–170.00)	157.00 ± 10.44 158.00 (151.00–163.31)	0.531	157.26 ± 11.72 158.00 (148.50–165.00)	160.82 ± 12.34 160.25 (152.50–170.50)	0.420
BMI, kg/m^2^	Mean±SD Median (IQR)	19.44 ± 3.63 18.72 (16.89–21.10)	19.51 ± 3.71 18.60 (16.89–21.20)	19.21 ± 3.50 18.71 (16.79–21.10)	0.513	18.21 ± 2.20 18.13 (16.60–19.80)	25.43 ± 3.22 24.60 (23.50–26.31)	**0.011**
Nutritional status by BMI	Underweight	3.4%	4.7%	2.4%	0.465	4.1%	0.0%	**<0.001**
	Normal	79.8%	78.4%	81.0%		95.9%	0.0%	
	Overweight	13.1%	12.3%	13.8%		0.0%	78.1%	
	Obesity	3.7%	4.7%	2.9%		0.0%	21.9%	
Body fat category	Underweight	46.4%	56.5%	38.3%	**0.003**	55.9%	0.0%	**<0.001**
	Normal	36.7%	30.0%	42.1%		38.7%	26.6%	
	Overweight	9.8%	6.5%	12.4%		5.4%	31.3%	
	Obesity	7.1%	7.1%	7.2%		0.0%	42.2%	
Hip circumference, cm	Mean±SD Median (IQR)	84.40 ± 9.92 84.00 (77.00–91.00)	83.51 ± 10.22 83.00 (76.0–90.0)	85.13 ± 9.63 86.00 (79.0–91.0)	0.441	82.12 ± 8.43 83.00 (76.0–88.0)	95.67 ± 9.06 96.00 (89.5–102.0)	0.140
WC, cm	Mean±SD Median (IQR)	65.69 ± 8.64 64.00 (60.00–70.00)	67.81 ± 9.24 66.00 (62.00–72.00)	63.96 ± 7.72 63.00 (59.00–68.00)	0.630	63.05 ± 5.78 63.00 (59.00–67.00)	78.73 ± 8.66 78.00 (72.75–85.50)	0.052
Abdominal obesity by WC	Yes	11.1%	11.7%	10.5%	0.718	1.6%	58.7%	**<0.001**
	No	88.9%	88.3%	89.5%		98.4%	41.3%	
WHR	Mean±SD Median (IQR)	0.78 ± 0.07 0.77 (0.74–0.82)	0.81 ± 0.07 0.81 (0.77–0.84)	0.75 ± 0.05 0.75 (0.71–0.79)	0.889	0.77 ± 0.06 0.77 (0.73–0.81)	0.82 ± 0.07 0.83 (0.78–0.85)	0.256
Abdominal obesity by WHR	Yes	6.0%	5.8%	6.2%	**<0.001**	4.1%	15.6%	**<0.001**
	No	94.0%	94.2%	93.8%		95.9%	84.4%	
WHtR	Mean±SD Median (IQR)	0.42 ± 0.05 0.41 (0.38–0.44)	0.43 ± 0.05 0.42 (0.39–0.45)	0.41 ± 0.05 0.40 (0.38–0.43)	**0.043**	0.40 ± 0.03 0.40 (0.38–0.42)	0.49 ± 0.04 0.48 (0.45–0.52)	**0.043**
Abdominal obesity by WHtR	Yes	8.1%	10.5%	6.2%	**<0.001**	0.6%	45.3%	**<0.001**
	No	91.9%	89.5%	93.8%		99.4%	54.7%	
SBP category	Normal	72.7%	66.7%	77.6%	**0.003**	76.3%	54.7%	**0.005**
	Normal high	8.4%	6.4%	10.0%		7.6%	12.5%	
	Stage 1 HTN	9.4%	14.0%	5.7%		8.2%	15.6%	
	Stage 2 HTN	9.4%	12.9%	6.7%		7.9%	17.2%	
DBP category	Normal	71.7%	66.1%	76.2%	0.100	73.5%	62.5%	**0.043**
	Normal high	9.2%	12.3%	6.7%		8.2%	14.1%	
	Stage 1 HTN	9.7%	9.9%	9.5%		8.2%	17.2%	
	Stage 2 HTN	9.4%	11.7%	7.6%		10.1%	6.3%	

BMI, body mass index; DBP, diastolic blood pressure; HTN, hypertension; IQR, interquartile range; M, mean; *N*, number; SBP, systolic blood pressure; SD, standard deviation; WC, waist circumference; WHR, waist-to-hip ratio; WHtR, waist-to-height ratio. Statistically significant values are in bold with the significance level set at *p* < 0.05. Data are presented as % of participants unless otherwise indicated. The χ^2^ Pearson test (categorical variables) or Wilcoxon rank-sum test (continuous variable) were applied.

There was substantial agreement between the overweight status assessed by BMI and the percentage of body fat (91.03%; *κ* = 0.68, *p* < 0.001). Only 34 respondents were classified differently using BMI and body fat percentage. There was moderate agreement between BMI and WHtR (90.29%; *κ* = 0.56, *p* < 0.001; 37 respondents classified differently), but only slight-to-fair agreement between BMI and WHR (82.41%; κ = 0.16, *p* = 0.001; 67 respondents differently classified) or WHtR and WHR (90.03%; *κ* = 0.24, *p* < 0.001; 38 respondents classified differently).

The main characteristics of participants with and without overweight and obesity are presented in Table 2. Respondents with overweight and obesity had significantly higher SBP and DBP compared with normal-weight and underweight individuals.

### 3.2 Associations of selected features with excess body mass (BMI) and abdominal obesity (WHtR)

There was no association between the intake of selected products and beverages and the risk of excess body mass (by BMI) or abdominal obesity (by WHtR), except for the finding that respondents who more often drank cola-type products were less likely to have abdominal obesity (Supplementary Table S1). The general nutritional indices did not differ between participants with overweight and obesity, those with abdominal obesity, and the remaining respondents. After adjusting for the characteristics of respondents, a significant correlation was revealed for the DQI category. Respondents who reported a healthier diet were more likely to have abdominal obesity. More frequent snacking was associated with excess body mass and abdominal obesity in the study group. Adolescents may snack as a response to body and weight dissatisfaction, often skipping meals and replacing them with high caloric products. In addition, excess body mass was significantly correlated with satisfaction with one’s figure (respondents who were satisfied with their figure were less likely to be overweight or obese, or to have abdominal obesity), fear of becoming obese (respondents who feared becoming obese were more likely to have excess body mass, or to have abdominal obesity), exercising after eating to avoid weight gain (respondents who exercised after eating were more likely to have excess body mass, or to have abdominal obesity), being on a weight-loss diet currently or in the past (respondents who were on a weight-loss diet were more likely to have excess body mass, or to have abdominal obesity).

**Figure 1:**
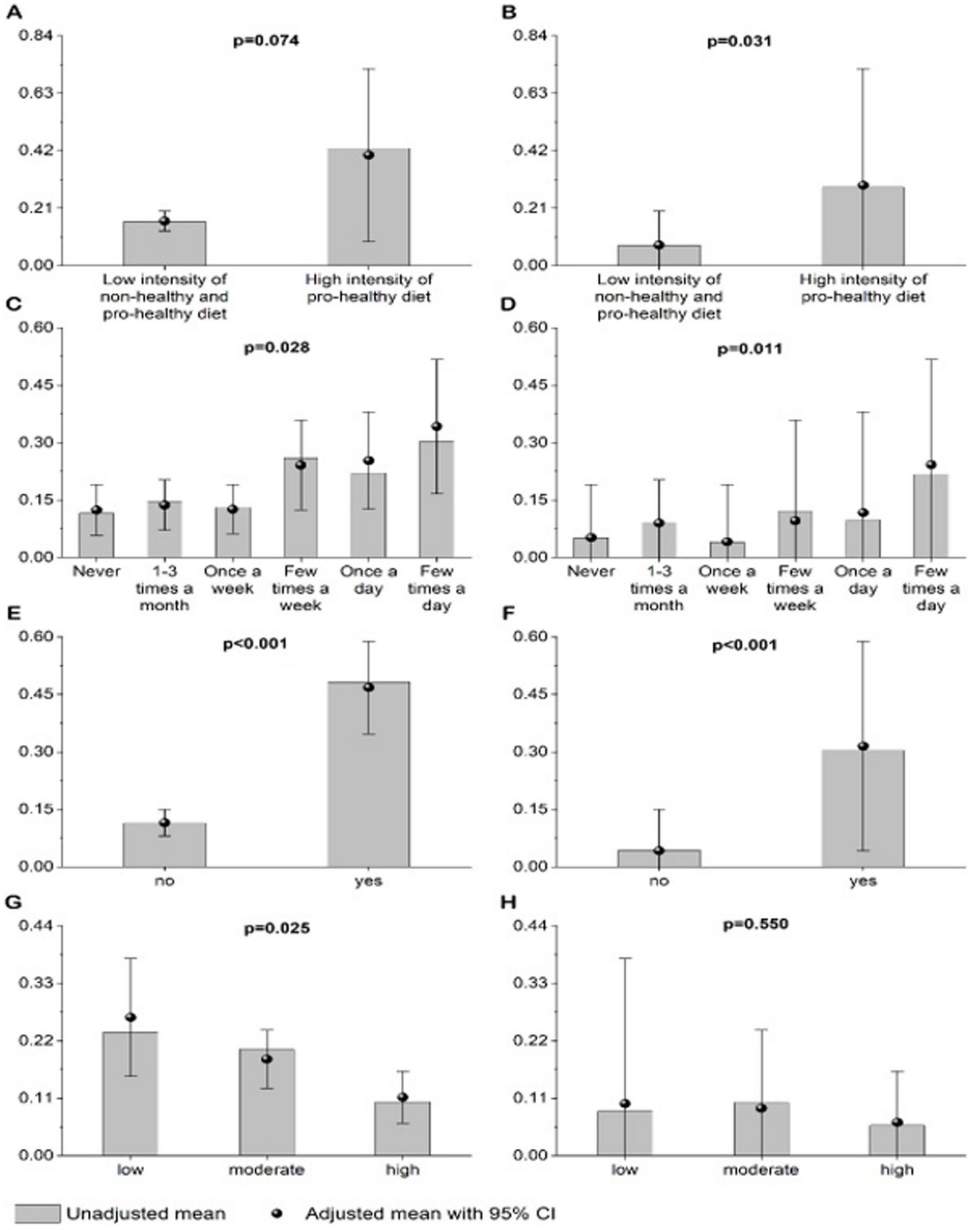
Unadjusted and adjusted (age, sex, SBP, DBP, place of living, number of siblings, parental work status, and self-reported financial situation) probability of being overweight or obese by BMI **(A,C,E,G)** in relation to: DQI category **(A)**, frequency of snacking **(C)**, any weight-loss diet **(E)**, and level of physical activity at school **(G)** or probability of having abdominal obesity by WHtR **(B,D,F,H)** in relation to: DQI category **(B)**, frequency of snacking **(D)**, any weight-loss diet **(F)**, and level of physical activity at school **(H)**. *p* value < 0.05 was considered significant **(B–G)**.

**Figure 2:**
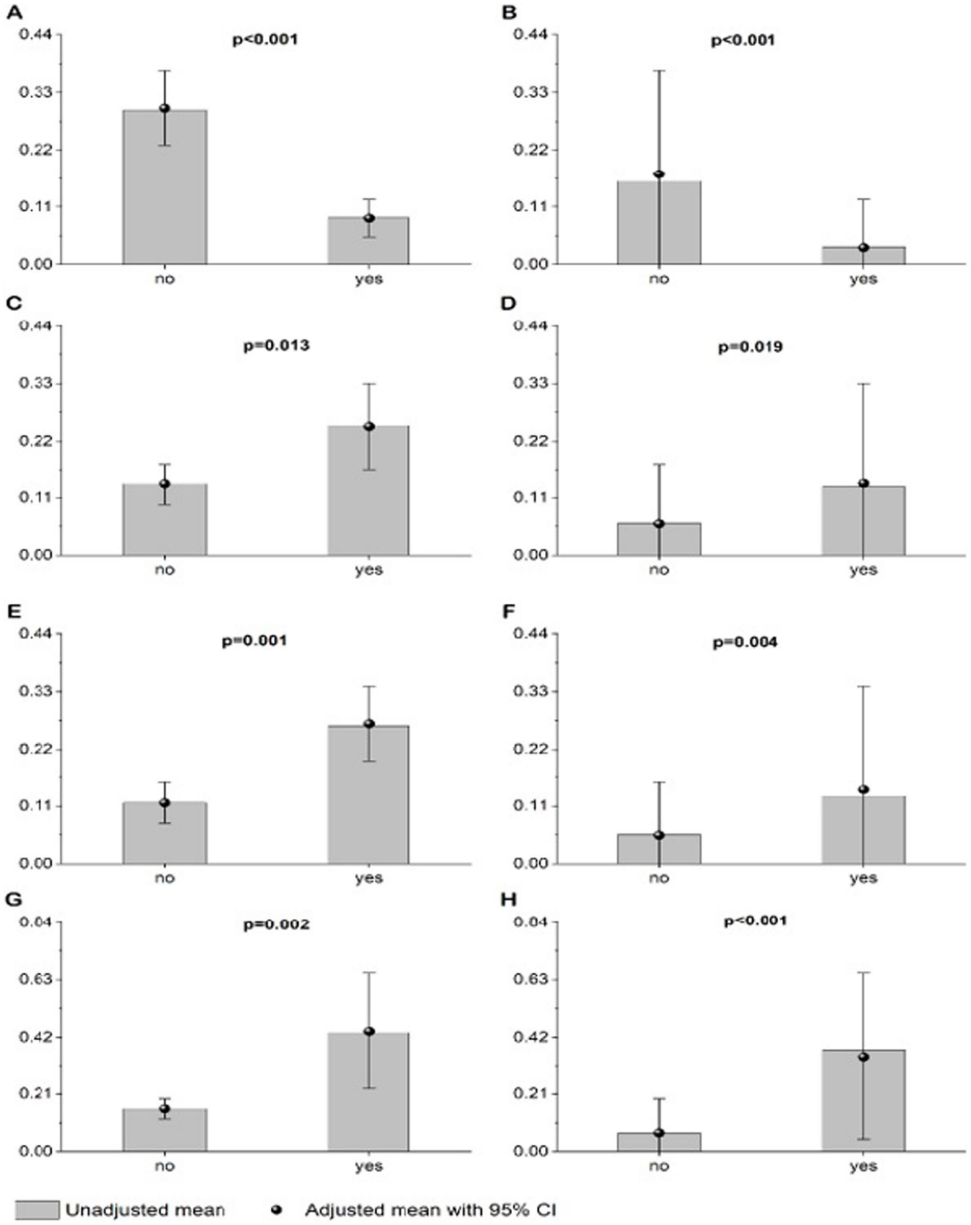
Unadjusted and adjusted (age, sex, SBP, DBP, place of living, number of siblings, parental work status, and self-reported financial situation) probability of being overweight or obese by BMI **(A,C,E,G)** in relation to: satisfaction with one’s figure **(A)**, fear of becoming obese **(C)**, receiving comments about one’s appearance **(E)**, and accepting one’s figure but starting a weight-loss diet because of pressure from the environment, mass media, etc. **(G)** or probability of having abdominal obesity by WHtR **(B,D,F,H)** in relation to: satisfaction with one’s figure **(B)**, fear of becoming obese **(D)**, receiving comments about onc’s appearance **(F)**, and accepting onc’a figure but starting a weight-loss dict because of pressure from the environment, mass media, etc. **(H)**. p value < 0.05 was considered significant **(A–G)**.

Respondents who received comments from others about their appearance were more likely to be overweight or obese, or to have abdominal obesity. In addition, a higher probability of having excess body mass or abdominal obesity was reported for respondents who started a weight-loss diet despite accepting their figure. Overweight or obesity was also significantly correlated with the opinion of respondents that “thinness is trendy” and that “a slim figure guarantees success in life.” Respondents who perceived their own figure as overweight or obese, those who perceived overweight figure as ideal, and those who were on any diet were significantly more likely to have excess body mass or abdominal obesity. Finally, excess body mass was significantly correlated with the level of physical activity at school (respondents with a higher level of physical activity at school were less likely to be overweight or obese) (Figures 1, 2).

### 3.3 Full logistic regression: BMI

The full logistic regression model taking into account the characteristics of respondents and other collected data revealed that the overweight or obesity status (BMI) was significantly associated with the child’s age, SBP, frequency of snacking, satisfaction with one’s figure, being on a weight-loss diet currently or in the past, receiving comments from others about one’s appearance, selected categories of the number of hours spent in front of the television or computer, and a high level of self-assessed physical activity at school (Table 3).

**Table 3:** Associations between the probability of being overweight or obese (BMI), characteristics of respondents, and self-reported nutritional or social behavior.

Variable		OR (95% CI)	*p*
Age	Per 1-year increase	0.71 (0.56 to 0.91)	**0.007**
SBP	Per 1-mmHg increase	1.07 (1.03 to 1.10)	**<0.001**
DBP	Per 1-mmHg increase	0.96 (0.93 to 1.002)	0.066
Snacking	Never	*Reference*	-
	1–3 times a month	1.30 (0.48 to 3.52)	0.611
	Once a week	2.26 (0.71 to 7.15)	0.165
	Few times a week	3.88 (1.38 to 10.88)	**0.010**
	Once a day	2.44 (0.76 to 7.86)	0.135
	Few times a day	8.17 (1.80 to 37.11)	**0.006**
	*Overall effect*	*-*	**0.046**
Satisfaction with one’s figure	No	*Reference*	-
	Yes	0.34 (0.16 to 0.70)	**0.003**
Being on a weight-loss diet currently or in the past	No	*Reference*	-
	Yes	7.75 (3.54 to 16.97)	**<0.001**
Receiving comments from others about one’s appearance	No	*Reference*	-
	Yes	3.12 (1.54 to 6.34)	**0.002**
Hours per day in front of the television or computer	From 8 to 10 h	*Reference*	-
	From 6 to 8 h	0.08 (0.00 to 1.50)	0.091
	From 4 to 6 h	0.10 (0.01 to 0.90)	**0.040**
	From 2 to 4 h	0.11 (0.01 to 0.85)	**0.034**
	Less than 2 h	0.15 (0.02 to 1.23)	0.077
	*Overall effect*	*-*	0.242
Physical activity at school	Low	*Reference*	-
	Moderate	0.77 (0.34 to 1.74)	0.533
	High	0.38 (0.15 to 0.94)	**0.036**
	*Overall effect*	*-*	0.073

Logistic regression; *N* = 381; log pseudolikelihood = −117.17; intercept (baseline odds) = 0.31 (95% CI: 0.004 to 24.58). Statistically significant values are in bold, with the significance level set at *p* < 0.05.

### 3.4 Full logistic regression: WHtR

The full logistic regression model revealed that abdominal obesity (based on WHtR) was significantly associated with the respondent’s age, SBP, DBP, self-reported financial status, place of living, satisfaction with one’s figure, exercising after eating to avoid weight gain, and being on a weight-loss diet currently or in the past (Table 4).

**Table 4:** Associations between the probability of abdominal obesity (based on WHtR), characteristics of respondents, and self-reported nutritional or social behavior.

Variable		OR (95% CI)	*p*
Age	Per 1-year increase	0.37 (0.22 to 0.61)	**<0.001**
Sex	Boys	*Reference*	-
	Girls	0.24 (0.05 to 1.19)	0.080
SBP	Per 1-mmHg increase	1.10 (1.04 to 1.17)	**0.001**
DBP	Per 1-mmHg increase	0.95 (0.91 to 0.999)	**0.045**
Self-reported financial status (having enough money to buy food)	Never	*Reference*	-
	Sometimes	1.38 (0.11 to 16.91)	0.803
	Most of the time	0.02 (0.0005 to 1.32)	0.069
	Always	3.93 (0.43 to 35.81)	0.225
	*Overall effect*	*-*	**0.009**
Place of living	Village	*Reference*	-
	Town	43.39 (2.28 to 827.07)	**0.012**
	City	0.47 (0.10 to 2.24)	0.343
	*Overall effect*	*-*	**0.033**
Satisfaction with one’s figure	No	*Reference*	-
	Yes	0.13 (0.02 to 0.74)	**0.021**
Exercising after eating to avoid weight gain	No	*Reference*	-
	Yes	4.53 (1.22 to 16.80)	**0.024**
Being on a weight-loss diet currently or in the past	No	*Reference*	-
	Yes	11.99 (1.09 to 131.86)	**0.042**

Logistic regression; *N* = 381; log pseudolikelihood = −51.07; intercept (baseline odds) = 0.0148 (95% CI: 0.00003 to 6.4492). Statistically significant values are in bold, with the significance level set at *p* < 0.05.

## 4 Discussion

The high prevalence of overweight and obesity in children and adolescents remains a significant concern. In our study, excess body mass was reported in almost 17% of male and female participants. According to the World Health Organization, excess body mass (including obesity) in children and adolescents aged 5–19 years increased from 8 to 20% in the last 30 years (1990–2022) (1). A previous study reported the dual burden of underweight and overweight in young adolescents across 21 low- and middle-income countries, with differences in prevalence across regions (33). According to a recent national survey conducted during the COVID-19 pandemic among early-school children in Poland, the overall prevalence of overweight and obesity was 35.6% (38.8% of boys and 32.2% of girls) (34). The most recent Health Behavior in School-aged Children (HBSC) study reported overweight and obesity in 41% of 11-year-old boys and 19% of 11-year-old girls, 35% of 13-year-old boys and 18% of 13-year-old girls, and 28% of 15-year-old boys, and 13% of 15-year-old girls (35). This confirms the significant impact of COVID-19 restrictions on the prevalence of overweight and obesity among children and adolescents.

WC and WHtR can be used as noninvasive and inexpensive markers of abdominal obesity and cardiometabolic risk in children and adolescents (25). WHtR measurement may be considered in regular health check-ups of children and adolescents to assess cardiovascular risk regardless of BMI (13). WHtR has gained increasing interest as a measure of abdominal obesity, with a simple public health message: “keep your waist circumference below half your height” (36). Therefore, due to its methodological adequacy and practical applicability, WHtR was used in the model. The overall prevalence of abdominal obesity based on WHtR was 8.1% (10.5% in boys, 6.2% in girls). In our previous study in adolescents aged 16–18 years from Krakow, abdominal obesity was reported in 15.5, 10.7, and 21.7% of participants based on WC, WHtR, and WHR, respectively (37). This contrasts with our current findings, in which abdominal obesity was identified in 11.1% of participants based on WC and in only 6% based on WHR. These results do not support the increasing rates of abdominal obesity previously reported by Suder et al. in Polish schoolchildren aged 7–18 years, who found a higher prevalence of abdominal obesity than excess BMI between 1966 and 2012 (38). In comparison, the prevalence of abdominal obesity measured by WC was 15.9% among adolescents in Germany, 14.8% in Italy, and 12.7% in Norway. Overall, 15.4% of participants from these countries were affected (39), indicating a higher prevalence than observed in our study.

Sleep, low physical activity, and a sedentary lifestyle are known contributing factors to overweight and obesity in children and adolescents (7, 15, 16). These findings are consistent with our current study and also with our previous research showing that low physical activity is associated with a higher risk of general and abdominal obesity (37). A significant association between physical activity and short sleep duration and abdominal obesity was reported in children and adolescents (40). Sedentary habits, such as watching television, are linked to childhood obesity through mechanisms such as displacement of physical activity, unhealthy food preferences, higher energy intake, and overconsumption due to distraction. Less time spent in front of a television or computer screen is associated with lower risk of overweight and obesity. Interventions aimed at reducing sedentary behaviors in children, such as replacing sedentary time with moderate-to-vigorous physical activity, are associated with a reduction in visceral adipose tissue and fat mass percentage (41, 42).

Low self-esteem and distorted body image was associated with abnormal weight in children and adolescents (43, 44). Our current findings reflect adolescents’ perceptions of their body shape and weight, underscoring the importance of psychological factors in the prevention of overweight and obesity, including abdominal obesity. Results from the international HBSC survey indicated that Polish adolescents report the lowest self-esteem among the participating countries (35). A British study showed that the likelihood of emotional and externalizing symptoms in boys and girls was higher with an increase in BMI z-score, which was linked to dissatisfaction with appearance and low self-esteem in adolescence (45). So-called “emotional eating,” defined as the use of food to cope with stress and negative feelings, was observed among adolescents. Emotional eating was associated with overeating and loss of control over food behaviors. High intake of high-calorie, low-nutrient-density foods was also common, resulting in excess body mass (46). A strong relationship between excess body mass and attitudes toward excess body mass and self-esteem in adolescents could be part of the observed problem of internal bias toward body mass. This is a process in which people adopt and believe in the negative stereotypes about their body mass, which can lead to feelings of shame and low self-esteem. These people may believe that they are personally responsible for their own body mass (excluding the influence of other factors), which can cause low self-esteem, depressive and anxiety disorders, or even eating disorders (47).

In our study, the DQI indicated a neutral dietary effect in each of the study groups. The frequency of consumption of foods with potentially adverse health effects was similar to that of foods with potentially beneficial health effects. The observed paradox, in which adolescents with a higher intensity of health-promoting diet according to the DQI had a higher risk of excess body mass, can be explained by an attempt to improve their behavior out of fear of being overweight. This seems to be in line with the British study, in which participants who attempted to restrict food intake or exercise were more likely to gain weight in later adolescence (45). In a study of 190,296 students with a mean age of 13.3 ± 1.0 years, normal-weight and overweight or obese adolescents who misperceived their weight showed less healthy snacking patterns. On the other hand, underweight students who misperceived their weight showed healthier snacking patterns (48).

A healthy dietary pattern with a high intake of fruit, vegetables, fish, milk, and dairy products is associated with a lower risk of overweight and obesity. In contrast, this risk is increased in individuals who consume large amounts of fast food, sugar, sweets, and sweetened beverages, skip breakfast, and smoke (49). Risk scores based on variables such as biological sex, age, maternal BMI, active commuting, soft drink consumption, and weight have been developed to accurately predict abdominal obesity in adolescents (50, 51). The percentage of energy derived from dietary fat intake was strongly associated with total and abdominal adiposity, independent of physical activity in adolescents (52). Moreover, a high-fat diet, together with low carbohydrate and protein intake, was found to be associated with obesity in children and adolescents, suggesting the importance of a healthy, low-fat diet in the prevention of abdominal obesity (53). Preventive interventions that combine lifestyle factors seem to have additional benefits over isolated interventions based exclusively on diet or physical activity (54).

In this study, we aimed to identify factors associated with the development of overweight and obesity, including abdominal obesity. A key strength of the study was the measurement a wide range of parameters related to excess body mass, including abdominal obesity. In addition, we used a questionnaire that included both nutritional and non-nutritional items, such as self-reported weight and attitudes toward overweight and obesity. This research specifically focused on rural adolescents, which distinguishes it further, as the relationship between urbanization and excess body mass is already well established in the literature. However, our study has several limitations. The primary limitation is its cross-sectional design, which precludes the establishment of causal relationships between obesity and the variables assessed. To explore causality, a longitudinal study may be considered in the future. The response rate at the school, class, and student level was not calculated. Additionally, the study was based on data collected between 2016 and 2019, which may limit the relevance of the findings to the current context. Inferential analysis was further constrained by significant differences in group sizes, which predominantly influenced effect size estimates. The study employed convenience sampling, and the potential precision of the estimates was addressed by calculating the proportions of children with obesity, overweight, or underweight based on BMI z-scores with 95% CIs calculated using the Agresti-Coull method. However, the post-hoc power analysis was not performed, as it was expected to be low in studies of this kind. The relatively low incidence of diagnosed respondents with excess body mass, particularly abdominal obesity, resulted in poor fit of the logistic regression model. Thus, the results of the logistic regression analysis should be interpreted with caution, although they confirm the previously reported associations between selected risk factors and body mass disorders. Finally, although both WC and WHtR are recognized as reliable markers of metabolic comorbidities and are suitable for assessing the prevalence of abdominal obesity, the use of WHtR alone in the model may be considered a limitation of the study. As an exploratory study, these findings require confirmation in future research.

## 5 Conclusion

In conclusion, the risk of excess body mass, including abdominal obesity, in adolescents is influenced by a combination of lifestyle factors, but also by self-esteem and environmental influences, highlighting the importance of early intervention and targeted lifestyle modification. It is important to identify modifiable risk factors to prevent obesity, including abdominal obesity, in adolescents. Excess body mass is associated with multiple factors, the influence of which can fluctuate over time, particularly in relation to changing conditions and environmental pressures, such as the mass media, even in less urbanized areas. Therefore, further research is needed in the adolescent population to develop appropriate interventions and mitigate the risk of excess body mass.

## Data Availability

The raw data supporting the conclusions of this article will be made available by the authors, without undue reservation.
