# Supplementary material for: Dietary and non-dietary risk factors associated with excess body mass and abdominal obesity in adolescents from rural regions of southern Poland: a cross-sectional study
Source: Front Public Health. 2025 Jun 18;13:1578906. doi: 10.3389/fpubh.2025.1578906 (PMC12213464; doi:10.3389/fpubh.2025.1578906)
Supplement: Supplementary file 1 [file Data_Sheet_1.pdf]

Evaluation of nutritional status on the basis of a body composition analysis in children from the Małopolska region - self-administered questionnaire

Dear Student,

We are conducting a scientific study in which one of the elements is to find out the dietary behavior and attitudes towards one's own figure and the diets of young people. The information provided is anonymous and confidential and will only be used for scientific purposes by the Department of Human Nutrition, Institute of Public Health, Jagiellonian University Medical College.

Thank you in advance for your time and reliable answers.

Instruction: please read the questions and mark an X for one or more answers, as instructed next to the question.

Date of survey (day, month, year) □□ □□ □□□□ Respondent code □□□

Part A. Dietary habits over the last year

1. How many meals do you usually consume daily?
Please give one answer.
Meal - any of the regular occasions in a day when a reasonably large amount of food is eaten, e.g. morning, noon, evening.
(1)__ 1 meal
(2)__ 2 meals
(3)__ 3 meals
(4)__ 4 meals
(5)__ 5 meals or more

2. Do you consume meals at regular times?
Please give one answer.
(1)__ No
(2)__ Yes, but only some of them
(3)__ Yes, all of them

3. How often do you snack between the meals?
Please give one answer.
Snacking – usually a small portion of food eaten occasionally between the meals.
(1)__ Never
(2)__ 1-3 times a month
(3)__ Once a week
(4)__ Few times a week
(5)__ Once a day
(6)__ Few times a day

4. What types of food do you usually consume between the meals during the weekdays?
You can give more than one answer.
(10/1)__ Fruit
(10/2)__ Vegetables
(10/3)__ Unsweetened dairy beverages and desserts, e.g. yoghurts, curd/cream cheese, milk
(10/4)__ Sweetened dairy beverages and desserts, e.g. homogenized cheese, sweetened milk beverages, flavored milk
(10/5)__ Sweet snacks, e.g. confectionary, biscuits, cakes, chocolate bars, cereal bars, wafers
(10/6)__ Savory snacks, e.g. crackers, pretzels, crisps, potato chips
(10/7)__ Nuts, almonds, seeds
(10/8)__Other, please list …………………………

Part B. Food frequency consumption - We would like to learn, how often do you consume these foods? While answering to the questions, please consider foods eaten over the last year during your meals and snacking, eaten at home and away.
In this part please give only one answer to each question.

5. How often do you consume the products listed below?
(For each product in the table, insert one X in the space provided)

Product	Few times a day	Once a day	Few times a week	Once a week	1-3 times a month	Never
White bread and bakery products, e.g. bread rolls						
Wholemeal (brown) bread/bread rolls						
White rice, white pasta, fine-ground groats						
Buckwheat, oats, wholegrain pasta or other coarse-ground groats						
Fast foods, e.g. potato chips, hamburgers, pizza, hot-dogs						
Butter						
Milk (including flavored milk, hot chocolate)						
Fermented milk beverages, e.g. yoghurts, kefir (natural or flavored)						
Curd products, e.g. cottage cheese, homogenized cheese, fromage frais						
Cheese (including processed cheese, blue cheese)						
Cold meats, smoked sausages, hot-dogs						
Red meat, e.g. pork, beef, veal						
White meat, e.g. chicken, turkey, rabbit						
Fish						
Eggs						
Pulses-based foods, e.g. from beans, peas, soybeans, lentils						
Potatoes (excluding chips and crisps)						
Fruit						
Vegetables						
Sweets, e.g. confectionary, biscuits, cakes, chocolate bars, cereal bars, other						
Fruit juices						
Vegetable juices or fruit and vegetable juices						
Tea						
Coffee						
Sweetened carbonated or still such as Sprite, Fanta, lemonade						
Cola beverages such as Coca-Cola, Pepsi						
Energy drinks such as Red Bull						
Water, e.g. mineral, tap water						
Alcoholic beverages						

Part C. Attitudes towards one's own figure and diet

6. What are your attitudes towards your own body shape and diets?
(For each question in the table, place one X in the space provided)

Question	Yes	Not
Are you satisfied with your figure?		
Do you experience a strong fear of getting fat?		
Do you exercise after eating to keep from gaining weight?		
Have you ever followed a weight loss diet?		
Have you ever faced comments about your appearance from those close to you?		
Have you ever succumbed to peer pressure, mass media and decided to go on a weight-loss diet despite accepting your figure?		

7. What are your behaviors in relation to overweight and obesity?
(For each statement in the table, place one X in the space provided)

Type of statement	Completely disagree	Rather disagree	No opinion	Rather agree	Completely agree
A slim figure is a sign of good health					
An obese figure is unsightly but not detrimental to health					
Fat people have fewer friends					
An excessively lean figure is unsightly					
A very slim figure is indicative of undernutrition					
Obesity is a cause of disease					
Skinny is trendy					
Being overweight indicates a lack of concern for one's appearance					
A slim figure guarantees success in life					

8. Write the number of the silhouette that is most similar to yours in the images:………..

9. Write the number of the silhouette that you think is the most ideal in the figures:……….

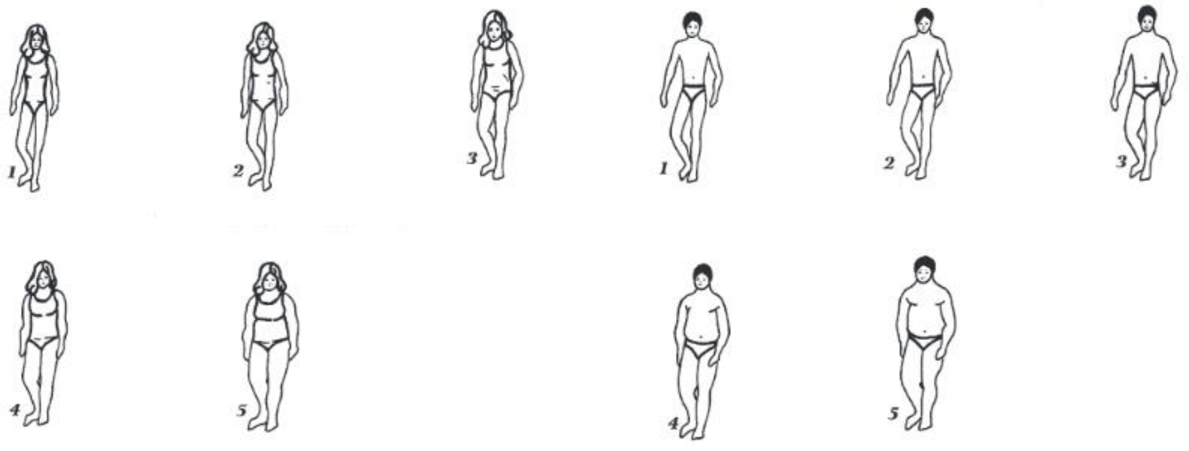

Part D. Lifestyle

We would like to ask you questions referring to your lifestyle and some personal data.
If you feel that some of the questions are too personal, you can refuse to answer. We would be however grateful for every honest answer given.
In this part please give one answer to each question.

10. Have you ever followed a diet?
(1)__ No
(2)__ Yes, as advised by my doctor for medical reasons
(3)__ Yes, it was my personal decision

11. How often do you eat out, e.g. in a bar, restaurant, café, canteen?
(1)__ Never
(2)__ 1-3 times a month
(3)__ Once a week
(4)__ Few times a week
(5)__ Once a day
(6)__ Few times a day

12 Have you ever tried smoked cigarettes or e-cigarettes?
(1)__ No
(2)__ Yes

13. Have you ever tried drinking alcohol?
(1)__ No
(2)__ Yes

14. How many hours do you sleep a day during weekdays?
(1)__ 6 or less hours/day
(2)__ more than 6, but less than 9 hours/day
(3)__ 9 or more hours/day

15. How many hours do you sleep a day during the weekend?
(1)__ 6 or less hours/day
(2)__ more than 6, but less than 9 hours/day
(3)__ 9 or more hours/day

16. How many hours a day (on average) do you spend watching TV or using a computer
(1)__ Less than 2 hours
(2)__ from 2 to almost 4 hours
(3)__ from 4 to almost 6 hours
(4)__ from 6 to almost 8 hours
(5)__ from 8 to almost 10 hours
(6)__ More than 10 hours

17. How would you describe your physical activity at school?
Present the ‘Show card No. 3’ to the respondent
(1)__ Low: more than 70% of time is sedentary
(2)__ Moderate: about 50% of time is sedentary and 50% active
(3)__ High: about 70% of time is active or physical labour of high intensity

18. How would you describe your physical activity during your time off?
(1)__ Low: mostly sedentary, watching TV, reading newspapers/books, light house works, walking for 1-2 hours/week
(2)__ Moderate: walking, cycling, exercise, gardening or other light physical activity for 2-3 hours/week
(3)__ High: cycling, running, gardening and other sport/recreational activities that require physical activity for longer than 3 hours/week

Part E. Personal data

19. Sex:
(1)__ Male
(2)__ Female

20. Date of birth: Day |__ | __ | Month |__ | __ | Year |__ | __ | __ | __ | enter e.g. 01.12.2005

21. What is your place of residence?
(1)__ Village
(2)__ Small town
(3)__ Provincial city

22. School:
(1)__ Primary
(2)__ Middle school

23. Do you have any siblings:
(1)__ No
(2)__ Yes, Sisters ….. Brother …..

24. Do your parents work professionally?
(1)__ No
(2)__ Yes, only mom
(3)__ Yes, only dad
(4) __ Yes both

25. Do parents always have enough money to buy the food they want to buy
(1)__ No
(2)__ Sometimes not
(3)__ Sometimes yes
(4) __ Yes

26. Do you have problems at school?
(1)__ No
(2) __ Yes - If yes, what do they concern …………………………………

Thank you for your participation in the survey and your time ☺
