# Supplementary material for: Dietary and non-dietary risk factors associated with excess body mass and abdominal obesity in adolescents from rural regions of southern Poland: a cross-sectional study
Source: Front Public Health. 2025 Jun 18;13:1578906. doi: 10.3389/fpubh.2025.1578906 (PMC12213464; doi:10.3389/fpubh.2025.1578906)
Supplement: Supplementary file 2 [file Table_1.docx]

Supplementary Material

# Supplementary Data

TABLE S1. Probability of being overweight or obese among respondents in relation to self-reported nutritional or societal behavior and own opinions on those aspects.

| Category | Subcategory | Overweight or obesity (BMI) | | | Abdominal obesity (WHtR) | | |
| --- | --- | --- | --- | --- | --- | --- | --- |
|  |  | Raw frequency, n/N (%) | Adjusted probability (95% CI) ^a^ | p-value ^b^ | Raw frequency, n/N (%) | Adjusted probability (95% CI) ^a^ | p-value ^b^ |
| DQI category | High intensity of nonhealthy dietary characteristics | 0/9 (0.0%) | excluded | 0.074 | 0/9 (0.0%) | excluded | **0.031** |
|  | Low intensity of nonhealthy and prohealthy dietary characteristics | 58/358 (16.2%) | 16.3% (12.7% to 19.9%) |  | 27/358 (7.5%) | 7.5% (4.9% to 10.1%) |  |
|  | High intensity of prohealthy dietary characteristics | 6/14 (42.9%) | 40.4% (8.7% to 72.0%) |  | 4/14 (28.6%) | 29.4% (1.0% to 57.8%) |  |
| Number of meals consumed a day | 1 | 0/2 (0.0%) | 14.2% (2.1% to 26.3%) | 0.673 | 0/2 (0.0%) | 4.3% (0.0% to 9.7%) | 0.272 |
|  | 2 | 1/12 (8.3%) | 15.1% (6.5% to 23.7%) |  | 0/12 (0.0%) | 5.4% (1.0% to 9.9%) |  |
|  | 3 | 17/118 (14.4%) | 16.0% (10.9% to 21.2%) |  | 7/118 (5.9%) | 6.8% (3.6% to 10.0%) |  |
|  | 4 | 38/191 (19.9%) | 17.0% (13.4% to 20.7%) |  | 20/191 (10.5%) | 8.5% (5.7% to 11.2%) |  |
|  | 5 | 8/58 (13.8%) | 18.1% (11.3% to 24.8%) |  | 4/58 (6.9%) | 10.4% (4.9% to 15.9%) |  |
| Problems at school | No | 50/321 (15.6%) | 15.6% (11.7% to 19.4%) | 0.110 | 25/321 (7.8%) | 7.8% (4.9% to 10.6%) | 0.551 |
|  | Yes | 14/60 (23.3%) | 23.9% (13.7% to 34.0%) |  | 6/60 (10.0%) | 10.4% (1.8% to 18.9%) |  |
| eating meals at regular times of the day | no | 19/119 (16.0%) | 16.0% (9.4% to 22.7%) | 0.225 | 8/119 (6.7%) | 6.6% (1.9% to 11.4%) | 0.263 |
|  | Yes (some of them) | 31/207 (15.0%) | 15.2% (10.3% to 20.1%) |  | 14/207 (6.8%) | 7.4% (3.9% to 10.8%) |  |
|  | yes (all) | 14/55 (25.5%) | 24.5% (13.9% to 35.1%) |  | 9/55 (16.4%) | 13.6% (4.9% to 22.2%) |  |
| Snacking (eating between meals) | Never | 9/78 (11.5%) | 12.4% (5.7% to 19.0%) | **0.028** | 4/78 (5.1%) | 5.2% (0.4% to 9.9%) | **0.011** |
|  | 1-3 times a month | 13/90 (14.4%) | 13.7% (7.1% to 20.2%) |  | 8/90 (8.9%) | 9.0% (3.3% to 14.7%) |  |
|  | Once a week | 13/99 (13.1%) | 12.6% (6.1% to 19.0%) |  | 4/99 (4.0%) | 4.1% (0.1% to 8.2%) |  |
|  | Few times a week | 13/50 (26.0%) | 24.1% (12.4% to 35.8%) |  | 6/50 (12.0%) | 9.6% (1.2% to 17.9%) |  |
|  | Once a day | 9/41 (22.0%) | 25.3% (12.6% to 38.0%) |  | 4/41 (9.8%) | 11.7% (2.6% to 20.8%) |  |
|  | Few times a day | 7/23 (30.4%) | 34.2% (16.7% to 51.7%) |  | 5/23 (21.7%) | 24.2% (10.5% to 37.9%) |  |
| Snacking on fruits | No | 14/91 (15.4%) | 15.7% (8.9% to 22.4%) | 0.713 | 6/91 (6.6%) | 7.2% (2.0% to 12.4%) | 0.704 |
|  | Yes | 50/290 (17.2%) | 17.2% (13.0% to 21.4%) |  | 25/290 (8.6%) | 8.4% (5.4% to 11.5%) |  |
| Snacking on vegetables | No | 50/294 (17.0%) | 17.2% (13.1% to 21.3%) | 0.741 | 22/294 (7.5%) | 7.8% (4.7% to 10.8%) | 0.628 |
|  | Yes | 14/87 (16.1%) | 15.7% (8.5% to 23.0%) |  | 9/87 (10.3%) | 9.3% (3.6% to 15.0%) |  |
| Snacking on unsweetened dairy drinks and desserts | No | 42/247 (17.0%) | 17.6% (13.1% to 22.1%) | 0.581 | 19/247 (7.7%) | 7.9% (4.6% to 11.2%) | 0.806 |
|  | Yes | 22/134 (16.4%) | 15.5% (9.5% to 21.4%) |  | 12/134 (9.0%) | 8.6% (4.2% to 12.9%) |  |
| Snacking on sweetened dairy drinks and desserts | No | 49/282 (17.4%) | 17.2% (13.1% to 21.4%) | 0.710 | 25/282 (8.9%) | 9.1% (5.9% to 12.3%) | 0.296 |
|  | Yes | 15/99 (15.2%) | 15.6% (8.4% to 22.8%) |  | 6/99 (6.1%) | 5.6% (0.8% to 10.3%) |  |
| Sweet snacks | No | 34/190 (17.9%) | 18.5% (12.9% to 24.1%) | 0.404 | 17/190 (8.9%) | 8.9% (4.9% to 12.8%) | 0.610 |
|  | Yes | 30/191 (15.7%) | 15.2% (10.3% to 20.1%) |  | 14/191 (7.3%) | 7.4% (3.7% to 11.1%) |  |
| salty snacks | No | 50/279 (17.9%) | 17.3% (13.1% to 21.5%) | 0.684 | 27/279 (9.7%) | 9.2% (6.0% to 12.3%) | 0.178 |
|  | Yes | 14/102 (13.7%) | 15.5% (8.5% to 22.6%) |  | 4/102 (3.9%) | 4.8% (0.4% to 9.2%) |  |
| Nuts, almonds, seeds, pits | No | 49/315 (15.6%) | 16.3% (12.3% to 20.3%) | 0.529 | 23/315 (7.3%) | 7.7% (4.8% to 10.6%) | 0.535 |
|  | Yes | 15/66 (22.7%) | 19.1% (10.9% to 27.3%) |  | 8/66 (12.1%) | 9.8% (3.5% to 16.1%) |  |
| Other snacks | No | 61/362 (16.9%) | 16.9% (13.2% to 20.5%) | 0.980 | 30/362 (8.3%) | 8.3% (5.6% to 11.0%) | 0.685 |
|  | Yes | 3/19 (15.8%) | 16.7% (2.0% to 31.3%) |  | 1/19 (5.3%) | 5.7% (-4.6% to 16.1%) |  |
| Bright bread | Never | 7/24 (29.2%) | 21.9% (7.1% to 36.8%) | 0.341 | 5/24 (20.8%) | 16.6% (2.0% to 31.2%) | 0.106 |
|  | 1-3 times a month | 1/12 (8.3%) | 8.8% (0.0% to 24.3%) |  | 0/12 (0.0%) | excluded |  |
|  | Once a week | 4/12 (33.3%) | 37.6% (6.6% to 68.5%) |  | 3/12 (25.0%) | 29.1% (3.0% to 55.2%) |  |
|  | Few times a week | 7/57 (12.3%) | 11.7% (3.1% to 20.3%) |  | 3/57 (5.3%) | 3.6% (0.0% to 8.3%) |  |
|  | Once a day | 19/98 (19.4%) | 19.6% (11.8% to 27.4%) |  | 8/98 (8.2%) | 8.8% (3.4% to 14.1%) |  |
|  | Few times a day | 26/178 (14.6%) | 15.3% (10.2% to 20.3%) |  | 12/178 (6.7%) | 7.4% (3.5% to 11.2%) |  |
| Wholemeal bread | Never | 9/61 (14.8%) | 14.7% (5.6% to 23.8%) | 0.160 | 6/61 (9.8%) | 9.4% (2.7% to 16.2%) | 0.311 |
|  | 1-3 times a month | 14/86 (16.3%) | 18.5% (10.3% to 26.7%) |  | 7/86 (8.1%) | 9.6% (2.8% to 16.4%) |  |
|  | Once a week | 9/56 (16.1%) | 17.3% (7.9% to 26.7%) |  | 2/56 (3.6%) | 3.7% (0.7% to 8.0%) |  |
|  | Few times a week | 9/86 (10.5%) | 10.0% (3.8% to 16.2%) |  | 6/86 (7.0%) | 7.0% (1.8% to 12.2%) |  |
|  | Once a day | 15/52 (28.8%) | 27.8% (16.3% to 39.2%) |  | 8/52 (15.4%) | 14.1% (5.2% to 23.0%) |  |
|  | Few times a day | 8/40 (20.0%) | 17.2% (6.1% to 28.3%) |  | 2/40 (5.0%) | 4.4% (0.4% to 11.2%) |  |
| White rice, plain pasta or small groats | Never | 3/17 (17.6%) | 19.7% (0.0% to 40.9%) | 0.968 | 2/17 (11.8%) | 10.5% (-1.8% to 22.9%) | 0.380 |
|  | 1-3 times a month | 12/86 (14.0%) | 15.4% (7.7% to 23.0%) |  | 6/86 (7.0%) | 8.0% (2.2% to 13.9%) |  |
|  | Once a week | 17/104 (16.3%) | 15.8% (8.7% to 23.0%) |  | 10/104 (9.6%) | 9.4% (3.9% to 14.8%) |  |
|  | Few times a week | 22/133 (16.5%) | 16.7% (10.7% to 22.7%) |  | 8/133 (6.0%) | 5.8% (1.8% to 9.9%) |  |
|  | Once a day | 7/26 (26.9%) | 22.6% (6.8% to 38.4%) |  | 5/26 (19.2%) | 17.7% (3.6% to 31.8%) |  |
|  | Few times a day | 3/15 (20.0%) | 18.6% (0.3% to 36.9%) |  | 0/15 (0.0%) | excluded |  |
| Buckwheat, oatmeal, whole wheat pasta or other groats | Never | 7/36 (19.4%) | 19.2% (6.3% to 32.1%) | 0.711 | 5/36 (13.9%) | 13.4% (2.8% to 24.0%) | 0.676 |
|  | 1-3 times a month | 15/105 (14.3%) | 14.4% (8.3% to 20.5%) |  | 10/105 (9.5%) | 10.1% (4.7% to 15.5%) |  |
|  | Once a week | 13/78 (16.7%) | 16.8% (8.1% to 25.6%) |  | 5/78 (6.4%) | 6.7% (0.7% to 12.7%) |  |
|  | Few times a week | 14/91 (15.4%) | 15.5% (8.3% to 22.7%) |  | 5/91 (5.5%) | 5.3% (0.9% to 9.8%) |  |
|  | Once a day | 13/55 (23.6%) | 23.8% (12.4% to 35.1%) |  | 5/55 (9.1%) | 8.3% (2.1% to 14.5%) |  |
|  | Few times a day | 2/16 (12.5%) | 12.5% (0.0% to 27.6%) |  | 1/16 (6.3%) | 6.3% (0.0% to 18.0%) |  |
| Fast food | Never | 14/41 (34.1%) | 29.4% (15.3% to 43.6%) | 0.102 | 8/41 (19.5%) | 14.9% (4.5% to 25.2%) | 0.541 |
|  | 1-3 times a month | 39/227 (17.2%) | 17.3% (12.5% to 22.0%) |  | 18/227 (7.9%) | 8.4% (4.8% to 11.9%) |  |
|  | Once a week | 7/48 (14.6%) | 17.4% (6.7% to 28.2%) |  | 4/48 (8.3%) | 10.2% (2.7% to 17.6%) |  |
|  | Few times a week | 2/40 (5.0%) | 6.7% (0.0% to 15.3%) |  | 0/40 (0.0%) | excluded |  |
|  | Once a day | 1/14 (7.1%) | 5.1% (0.0% to 15.8%) |  | 1/14 (7.1%) | 5.1% (0.0% to 15.8%) |  |
|  | Few times a day | 1/11 (9.1%) | 6.4% (0.0% to 19.2%) |  | 0/11 (0.0%) | excluded |  |
| Butter | Never | 2/14 (14.3%) | 15.2% (0.0% to 37.7%) | 0.989 | 1/14 (7.1%) | 8.4% (0.0% to 25.3%) | 0.713 |
|  | 1-3 times a month | 2/16 (12.5%) | 14.7% (0.0% to 32.6%) |  | 3/16 (18.8%) | 17.4% (0.0% to 35.8%) |  |
|  | Once a week | 2/16 (12.5%) | 12.4% (0.0% to 28.2%) |  | 1/16 (6.3%) | 6.9% (0.0% to 19.3%) |  |
|  | Few times a week | 9/59 (15.3%) | 16.4% (0.0% to 25.6%) |  | 3/59 (5.1%) | 5.8% (0.0% to 12.6%) |  |
|  | Once a day | 24/116 (20.7%) | 18.6% (11.6% to 25.7%) |  | 13/116 (11.2%) | 9.5% (4.3% to 14.7%) |  |
|  | Few times a day | 25/160 (15.6%) | 16.4% (10.9% to 21.9%) |  | 10/160 (6.3%) | 6.8% (2.8% to 10.9%) |  |
| Milk including flavored milk, cocoa | Never | 5/21 (23.8%) | 20.0% (3.7% to 36.3%) | 0.495 | 5/21 (23.8%) | 23.6% (7.5% to 39.7%) | 0.166 |
|  | 1-3 times a month | 11/58 (19.0%) | 18.6% (9.2% to 28.0%) |  | 6/58 (10.3%) | 10.7% (2.7% to 18.6%) |  |
|  | Once a week | 9/57 (15.8%) | 14.4% (4.9% to 23.9%) |  | 3/57 (5.3%) | 4.1% (0.0% to 9.4%) |  |
|  | Few times a week | 18/113 (15.9%) | 17.0% (10.3% to 23.7%) |  | 8/113 (7.1%) | 7.3% (3.3% to 11.4%) |  |
|  | Once a day | 13/97 (13.4%) | 13.0% (6.8% to 19.3%) |  | 7/97 (7.2%) | 7.3% (2.0% to 12.5%) |  |
|  | Few times a day | 8/35 (22.9%) | 26.7% (13.1% to 40.3%) |  | 2/35 (5.7%) | 7.1% (0.0% to 16.2%) |  |
| Fermented milk drinks | Never | 4/19 (21.1%) | 17.2% (2.1% to 32.2%) | 0.871 | 4/19 (21.1%) | 16.4% (2.5% to 30.4%) | 0.421 |
|  | 1-3 times a month | 12/88 (13.6%) | 14.1% (7.2% to 21.0%) |  | 6/88 (6.8%) | 6.7% (1.9% to 11.5%) |  |
|  | Once a week | 14/78 (17.9%) | 16.5% (8.7% to 24.2%) |  | 5/78 (6.4%) | 5.4% (0.5% to 10.3%) |  |
|  | Few times a week | 20/125 (16.0%) | 16.7% (10.1% to 23.3%) |  | 8/125 (6.4%) | 7.4% (2.6% to 12.1%) |  |
|  | Once a day | 9/46 (19.6%) | 22.8% (11.4% to 34.3%) |  | 4/46 (8.7%) | 10.2% (1.8% to 18.6%) |  |
|  | Few times a day | 5/25 (20.0%) | 17.8% (3.2% to 32.4%) |  | 4/25 (16.0%) | 14.0% (0.5% to 27.4%) |  |
| Curd cheeses, including homogenized cheeses | Never | 7/62 (11.3%) | 10.4% (3.5% to 17.3%) | 0.503 | 5/62 (8.1%) | 7.1% (1.6% to 12.6%) | 0.873 |
|  | 1-3 times a month | 18/116 (15.5%) | 16.3% (9.7% to 22.9%) |  | 7/116 (6.0%) | 6.6% (1.8% to 11.5%) |  |
|  | Once a week | 19/92 (20.7%) | 20.7% (12.9% to 28.6%) |  | 9/92 (9.8%) | 9.3% (3.9% to 14.6%) |  |
|  | Few times a week | 13/80 (16.3%) | 16.9% (8.4% to 25.4%) |  | 6/80 (7.5%) | 7.6% (1.7% to 13.5%) |  |
|  | Once a day | 4/22 (18.2%) | 17.2% (2.0% to 32.4%) |  | 3/22 (13.6%) | 13.5% (0.0% to 27.3%) |  |
|  | Few times a day | 3/9 (33.3%) | 30.7% (0.0% to 63.7%) |  | 1/9 (11.1%) | 15.6% (0.0% to 46.0%) |  |
| Cheese, including processed, mold cheese | Never | 10/56 (17.9%) | 16.6% (7.4% to 25.7%) | 0.839 | 6/56 (10.7%) | 10.4% (2.9% to 17.8%) | 0.362 |
|  | 1-3 times a month | 10/75 (13.3%) | 14.2% (6.3% to 22.0%) |  | 3/75 (4.0%) | 4.6% (0.0% to 9.6%) |  |
|  | Once a week | 17/70 (24.3%) | 21.7% (12.3% to 31.1%) |  | 11/70 (15.7%) | 13.9% (5.9% to 21.9%) |  |
|  | Few times a week | 17/112 (15.2%) | 16.3% (9.6% to 23.1%) |  | 7/112 (6.3%) | 6.5% (1.5% to 11.4%) |  |
|  | Once a day | 7/44 (15.9%) | 17.4% (7.5% to 27.4%) |  | 4/44 (9.1%) | 9.2% (1.5% to 16.9%) |  |
|  | Few times a day | 3/24 (12.5%) | 12.2% (0.0% to 24.4%) |  | 0/24 (0.0%) | excluded |  |
| Cold cuts, sausages, sausages | Never | 2/9 (22.2%) | 15.7% (0.0% to 46.3%) | 0.844 | 1/9 (11.1%) | 9.2% (0.0% to 32.8%) | 0.771 |
|  | 1-3 times a month | 7/45 (15.6%) | 14.5% (3.8% to 25.3%) |  | 3/45 (6.7%) | 7.3% (0.0% to 14.9%) |  |
|  | Once a week | 11/55 (20.0%) | 19.3% (8.5% to 30.1%) |  | 7/55 (12.7%) | 13.3% (4.2% to 22.3%) |  |
|  | Few times a week | 22/151 (14.6%) | 14.5% (9.4% to 19.5%) |  | 11/151 (7.3%) | 7.0% (3.1% to 10.9%) |  |
|  | Once a day | 15/79 (19.0%) | 20.8% (12.0% to 29.5%) |  | 7/79 (8.9%) | 9.3% (3.2% to 15.3%) |  |
|  | Few times a day | 7/42 (16.7%) | 18.5% (6.2% to 30.8%) |  | 2/42 (4.8%) | 4.8% (0.0% to 12.7%) |  |
| Red meat | Never | 8/41 (19.5%) | 18.9% (6.1% to 31.7%) | 0.852 | 4/41 (9.8%) | 9.9% (1.6% to 18.2%) | 0.236 |
|  | 1-3 times a month | 18/114 (15.8%) | 16.2% (9.4% to 23.0%) |  | 8/114 (7.0%) | 7.6% (2.5% to 12.7%) |  |
|  | Once a week | 18/99 (18.2%) | 17.5% (11.0% to 24.0%) |  | 9/99 (9.1%) | 7.8% (3.2% to 12.4%) |  |
|  | Few times a week | 13/93 (14.0%) | 13.9% (6.9% to 20.9%) |  | 6/93 (6.5%) | 6.7% (1.4% to 11.9%) |  |
|  | Once a day | 4/22 (18.2%) | 20.4% (4.1% to 36.7%) |  | 4/22 (18.2%) | 20.4% (5.7% to 35.0%) |  |
|  | Few times a day | 3/12 (25.0%) | 27.6% (4.3% to 50.8%) |  | 0/12 (0.0%) | excluded |  |
| white meat | Never | 1/18 (5.6%) | 5.2% (0.0% to 14.9%) | 0.587 | 1/18 (5.6%) | 5.7% (0.0% to 16.2%) | 0.954 |
|  | 1-3 times a month | 7/54 (13.0%) | 13.6% (5.0% to 22.3%) |  | 5/54 (9.3%) | 10.5% (3.4% to 17.6%) |  |
|  | Once a week | 18/107 (16.8%) | 17.0% (10.2% to 23.8%) |  | 10/107 (9.3%) | 8.5% (3.8% to 13.2%) |  |
|  | Few times a week | 32/156 (20.5%) | 20.1% (13.9% to 26.4%) |  | 13/156 (8.3%) | 8.3% (3.8% to 12.8%) |  |
|  | Once a day | 4/33 (12.1%) | 12.6% (2.1% to 23.2%) |  | 2/33 (6.1%) | 7.3% (0.0% to 16.0%) |  |
|  | Few times a day | 2/13 (15.4%) | 15.5% (0.0% to 33.2%) |  | 0/13 (0.0%) | excluded |  |
| Fishes | Never | 7/35 (20.0%) | 16.9% (5.5% to 28.3%) | 0.893 | 4/35 (11.4%) | 11.2% (2.2% to 20.2%) | 0.862 |
|  | 1-3 times a month | 18/124 (14.5%) | 15.3% (9.3% to 21.3%) |  | 9/124 (7.3%) | 6.9% (2.6% to 11.1%) |  |
|  | Once a week | 27/145 (18.6%) | 18.2% (12.3% to 24.1%) |  | 13/145 (9.0%) | 9.5% (5.1% to 13.8%) |  |
|  | Few times a week | 8/58 (13.8%) | 15.7% (5.1% to 26.4%) |  | 4/58 (6.9%) | 8.1% (0.7% to 15.5%) |  |
|  | Once a day | 2/12 (16.7%) | 12.8% (0.0% to 30.4%) |  | 1/12 (8.3%) | 4.6% (0.0% to 15.5%) |  |
|  | Few times a day | 2/7 (28.6%) | 30.2% (0.1% to 60.3%) |  | 0/7 (0.0%) | excluded |  |
| Eggs | Never | 4/16 (25.0%) | 22.3% (1.5% to 43.2%) | 0.387 | 3/16 (18.8%) | 15.2% (0.0% to 31.6%) | 0.172 |
|  | 1-3 times a month | 7/58 (12.1%) | 11.1% (3.9% to 18.3%) |  | 5/58 (8.6%) | 7.4% (2.4% to 12.4%) |  |
|  | Once a week | 27/127 (21.3%) | 21.9% (15.0% to 28.8%) |  | 15/127 (11.8%) | 12.7% (7.2% to 18.3%) |  |
|  | Few times a week | 18/140 (12.9%) | 13.6% (7.7% to 19.5%) |  | 5/140 (3.6%) | 3.7% (0.2% to 7.2%) |  |
|  | Once a day | 4/24 (16.7%) | 17.7% (3.1% to 32.2%) |  | 3/24 (12.5%) | 12.0% (1.2% to 22.7%) |  |
|  | Few times a day | 4/16 (25.0%) | 21.2% (2.4% to 40.1%) |  | 0/16 (0.0%) | excluded |  |
| Legume seeds | Never | 11/68 (16.2%) | 14.6% (7.3% to 21.9%) | 0.114 | 5/68 (7.4%) | 6.9% (1.7% to 12.2%) | 0.180 |
|  | 1-3 times a month | 25/169 (14.8%) | 16.0% (10.6% to 21.3%) |  | 14/169 (8.3%) | 9.2% (4.9% to 13.6%) |  |
|  | Once a week | 12/75 (16.0%) | 15.0% (6.5% to 23.5%) |  | 5/75 (6.7%) | 6.0% (1.0% to 11.0%) |  |
|  | Few times a week | 6/43 (14.0%) | 14.5% (4.9% to 24.0%) |  | 3/43 (7.0%) | 5.7% (0.0% to 11.8%) |  |
|  | Once a day | 6/18 (33.3%) | 29.8% (8.5% to 51.2%) |  | 4/18 (22.2%) | 25.0% (4.9% to 45.0%) |  |
|  | Few times a day | 4/8 (50.0%) | 49.9% (19.2% to 80.6%) |  | 0/8 (0.0%) | excluded |  |
| Potatoes | Never | 2/10 (20.0%) | 17.1% (0.0% to 44.8%) | 0.449 | 2/10 (20.0%) | 19.7% (0.0% to 45.0%) | 0.340 |
|  | 1-3 times a month | 1/15 (6.7%) | 5.8% (0.0% to 17.3%) |  | 0/15 (0.0%) | excluded |  |
|  | Once a week | 12/51 (23.5%) | 25.3% (14.5% to 36.1%) |  | 7/51 (13.7%) | 14.2% (5.0% to 23.4%) |  |
|  | Few times a week | 27/172 (15.7%) | 15.4% (10.3% to 20.5%) |  | 12/172 (7.0%) | 7.1% (3.5% to 10.8%) |  |
|  | Once a day | 17/108 (15.7%) | 16.2% (9.4% to 23.0%) |  | 9/108 (8.3%) | 7.9% (3.1% to 12.8%) |  |
|  | Few times a day | 5/25 (20.0%) | 21.5% (4.6% to 38.3%) |  | 1/25 (4.0%) | 4.9% (0.0% to 14.8%) |  |
| Fruits | Never | 3/5 (60.0%) | 36.7% (0.0% to 77.6%) | 0.836 | 3/5 (60.0%) | 40.6% (0.0% to 82.5%) | 0.151 |
|  | 1-3 times a month | 0/4 (0.0%) | excluded |  | 0/4 (0.0%) | excluded |  |
|  | Once a week | 3/18 (16.7%) | 17.2% (0.9% to 33.5%) |  | 1/18 (5.6%) | 3.8% (0.0% to 9.2%) |  |
|  | Few times a week | 15/97 (15.5%) | 16.1% (8.9% to 23.2%) |  | 7/97 (7.2%) | 8.1% (2.7% to 13.6%) |  |
|  | Once a day | 15/99 (15.2%) | 16.0% (8.9% to 23.2%) |  | 6/99 (6.1%) | 6.4% (1.5% to 11.3%) |  |
|  | Few times a day | 28/158 (17.7%) | 17.3% (11.7% to 23.0%) |  | 14/158 (8.9%) | 8.6% (4.4% to 12.8%) |  |
| Vegetables | Never | 3/6 (50.0%) | 31.5% (0.0% to 65.5%) | 0.394 | 3/6 (50.0%) | 35.8% (0.0% to 74.3%) | 0.087 |
|  | 1-3 times a month | 4/13 (30.8%) | 34.6% (11.2% to 57.9%) |  | 2/13 (15.4%) | 14.0% (0.0% to 30.3%) |  |
|  | Once a week | 4/27 (14.8%) | 17.2% (4.1% to 30.2%) |  | 3/27 (11.1%) | 11.4% (1.0% to 21.8%) |  |
|  | Few times a week | 19/116 (16.4%) | 16.3% (9.7% to 22.8%) |  | 4/116 (3.4%) | 3.0% (0.0% to 6.0%) |  |
|  | Once a day | 14/117 (12.0%) | 13.3% (7.3% to 19.4%) |  | 9/117 (7.7%) | 9.5% (4.6% to 14.5%) |  |
|  | Few times a day | 20/102 (19.6%) | 17.8% (10.5% to 25.0%) |  | 10/102 (9.8%) | 9.3% (3.5% to 15.1%) |  |
| Sweets | Never | 3/9 (33.3%) | 24.8% (0.0% to 54.3%) | 0.315 | 2/9 (22.2%) | 14.8% (0.0% to 34.7%) | 0.498 |
|  | 1-3 times a month | 9/29 (31.0%) | 29.7% (11.6% to 47.7%) |  | 5/29 (17.2%) | 16.6% (3.9% to 29.3%) |  |
|  | Once a week | 13/63 (20.6%) | 21.0% (10.5% to 31.5%) |  | 6/63 (9.5%) | 9.6% (2.6% to 16.7%) |  |
|  | Few times a week | 21/135 (15.6%) | 16.0% (10.3% to 21.6%) |  | 9/135 (6.7%) | 6.9% (2.7% to 11.1%) |  |
|  | Once a day | 10/86 (11.6%) | 12.7% (5.9% to 19.5%) |  | 6/86 (7.0%) | 7.2% (2.0% to 12.4%) |  |
|  | Few times a day | 8/59 (13.6%) | 12.5% (4.4% to 20.6%) |  | 3/59 (5.1%) | 4.8% (0.0% to 10.8%) |  |
| Fruit juices | Never | 3/15 (20.0%) | 18.8% (0.0% to 39.5%) | 0.775 | 3/15 (20.0%) | 23.0% (0.7% to 45.4%) | 0.241 |
|  | 1-3 times a month | 3/24 (12.5%) | 10.9% (0.0% to 23.3%) |  | 2/24 (8.3%) | 6.9% (0.0% to 15.5%) |  |
|  | Once a week | 9/41 (22.0%) | 18.8% (8.7% to 28.9%) |  | 5/41 (12.2%) | 9.1% (2.5% to 15.6%) |  |
|  | Few times a week | 21/113 (18.6%) | 21.0% (13.4% to 28.6%) |  | 9/113 (8.0%) | 10.2% (3.8% to 16.5%) |  |
|  | Once a day | 15/93 (16.1%) | 15.0% (8.4% to 21.6%) |  | 10/93 (10.8%) | 9.3% (3.9% to 14.7%) |  |
|  | Few times a day | 13/95 (13.7%) | 14.5% (7.3% to 21.7%) |  | 2/95 (2.1%) | 2.4% (0.0% to 5.7%) |  |
| Vegetable or vegetable and fruit juices | Never | 12/64 (18.8%) | 18.9% (10.1% to 27.7%) | 0.955 | 8/64 (12.5%) | 13.9% (5.7% to 22.0%) | 0.390 |
|  | 1-3 times a month | 11/80 (13.8%) | 14.0% (6.0% to 22.0%) |  | 6/80 (7.5%) | 7.3% (2.1% to 12.5%) |  |
|  | Once a week | 10/65 (15.4%) | 16.4% (8.0% to 24.7%) |  | 4/65 (6.2%) | 5.2% (0.0% to 11.0%) |  |
|  | Few times a week | 16/91 (17.6%) | 17.2% (9.7% to 24.8%) |  | 8/91 (8.8%) | 9.1% (3.4% to 14.9%) |  |
|  | Once a day | 7/45 (15.6%) | 15.4% (5.5% to 25.3%) |  | 4/45 (8.9%) | 9.0% (1.5% to 16.6%) |  |
|  | Few times a day | 8/36 (22.2%) | 20.2% (7.6% to 32.9%) |  | 1/36 (2.8%) | 2.9% (0.0% to 7.5%) |  |
| Tea | Never | 1/4 (25.0%) | 17.5% (0.0% to 60.9%) | 0.668 | 1/4 (25.0%) | 23.6% (0.0% to 76.4%) | 0.090 |
|  | 1-3 times a month | 3/15 (20.0%) | 22.4% (3.8% to 41.0%) |  | 0/15 (0.0%) | excluded |  |
|  | Once a week | 5/17 (29.4%) | 29.8% (9.0% to 50.6%) |  | 4/17 (23.5%) | 23.9% (7.4% to 40.4%) |  |
|  | Few times a week | 7/57 (12.3%) | 13.5% (4.3% to 22.8%) |  | 4/57 (7.0%) | 8.7% (0.7% to 16.7%) |  |
|  | Once a day | 20/119 (16.8%) | 17.4% (10.7% to 24.2%) |  | 11/119 (9.2%) | 10.0% (4.7% to 15.3%) |  |
|  | Few times a day | 28/169 (16.6%) | 15.8% (10.5% to 21.1%) |  | 11/169 (6.5%) | 5.8% (2.3% to 9.3%) |  |
| coffee | Never | 40/245 (16.3%) | 14.9% (10.8% to 18.9%) | 0.567 | 24/245 (9.8%) | 9.3% (6.0% to 12.5%) | 0.533 |
|  | 1-3 times a month | 9/54 (16.7%) | 22.1% (9.8% to 34.4%) |  | 2/54 (3.7%) | 5.2% (0.0% to 12.3%) |  |
|  | Once a week | 4/26 (15.4%) | 14.0% (0.0% to 28.3%) |  | 2/26 (7.7%) | 6.3% (0.0% to 16.3%) |  |
|  | Few times a week | 3/23 (13.0%) | 18.6% (2.0% to 35.1%) |  | 0/23 (0.0%) | excluded |  |
|  | Once a day | 5/24 (20.8%) | 25.5% (7.9% to 43.1%) |  | 3/24 (12.5%) | 15.3% (2.6% to 28.0%) |  |
|  | Few times a day | 3/9 (33.3%) | 30.2% (3.1% to 57.3%) |  | 0/9 (0.0%) | excluded |  |
| Carbonated and non-carbonated drinks (Fanta, lemonade) | Never | 8/34 (23.5%) | 20.4% (8.0% to 32.9%) | 0.910 | 6/34 (17.6%) | 15.8% (6.3% to 25.3%) | 0.335 |
|  | 1-3 times a month | 21/113 (18.6%) | 19.2% (11.7% to 26.7%) |  | 8/113 (7.1%) | 8.6% (2.5% to 14.7%) |  |
|  | Once a week | 14/77 (18.2%) | 17.2% (9.6% to 24.8%) |  | 10/77 (13.0%) | 10.5% (4.3% to 16.7%) |  |
|  | Few times a week | 13/92 (14.1%) | 14.4% (7.1% to 21.7%) |  | 5/92 (5.4%) | 5.1% (0.3% to 10.0%) |  |
|  | Once a day | 4/39 (10.3%) | 12.7% (1.4% to 24.0%) |  | 2/39 (5.1%) | 6.9% (0.0% to 15.4%) |  |
|  | Few times a day | 4/26 (15.4%) | 14.7% (1.8% to 27.6%) |  | 0/26 (0.0%) | excluded |  |
| Cola-type drinks | Never | 16/59 (27.1%) | 25.0% (14.3% to 35.8%) | 0.304 | 10/59 (16.9%) | 17.9% (9.1% to 26.6%) | **0.027** |
|  | 1-3 times a month | 22/131 (16.8%) | 16.8% (10.5% to 23.1%) |  | 9/131 (6.9%) | 8.0% (2.8% to 13.1%) |  |
|  | Once a week | 14/83 (16.9%) | 16.6% (9.0% to 24.1%) |  | 10/83 (12.0%) | 10.8% (4.7% to 16.9%) |  |
|  | Few times a week | 8/64 (12.5%) | 12.8% (4.9% to 20.7%) |  | 1/64 (1.6%) | 1.3% (0.0% to 3.9%) |  |
|  | Once a day | 1/28 (3.6%) | 4.7% (0.0% to 13.9%) |  | 1/28 (3.6%) | 3.3% (0.0% to 11.7%) |  |
|  | Few times a day | 3/16 (18.8%) | 20.4% (2.6% to 38.2%) |  | 0/16 (0.0%) | excluded |  |
| Energy drinks | Never | 43/252 (17.1%) | 15.9% (11.7% to 20.1%) | 0.516 | 24/252 (9.5%) | 8.8% (5.6% to 12.0%) | 0.169 |
|  | 1-3 times a month | 12/83 (14.5%) | 16.5% (8.6% to 24.3%) |  | 4/83 (4.8%) | 5.5% (0.4% to 10.7%) |  |
|  | Once a week | 4/25 (16.0%) | 23.5% (4.6% to 42.4%) |  | 3/25 (12.0%) | 18.7% (2.9% to 34.6%) |  |
|  | Few times a week | 2/15 (13.3%) | 15.2% (0.0% to 36.0%) |  | 0/15 (0.0%) | excluded |  |
|  | Once a day | 2/4 (50.0%) | 47.5% (6.6% to 88.5%) |  | 0/4 (0.0%) | excluded |  |
|  | Few times a day | 1/2 (50.0%) | 30.2% (0.0% to 79.2%) |  | 0/2 (0.0%) | excluded |  |
| Mineral water | Never | 0/9 (0.0%) | excluded | 0.188 | 0/9 (0.0%) | excluded | 0.112 |
|  | 1-3 times a month | 4/33 (12.1%) | 12.8% (1.6% to 23.9%) |  | 1/33 (3.0%) | 4.0% (0.0% to 10.2%) |  |
|  | Once a week | 5/18 (27.8%) | 30.9% (11.1% to 50.8%) |  | 0/18 (0.0%) | excluded |  |
|  | Few times a week | 5/51 (9.8%) | 10.3% (1.4% to 19.3%) |  | 2/51 (3.9%) | 3.0% (0.0% to 7.3%) |  |
|  | Once a day | 8/63 (12.7%) | 12.6% (5.3% to 19.9%) |  | 4/63 (6.3%) | 5.9% (1.0% to 10.7%) |  |
|  | Few times a day | 42/207 (20.3%) | 19.9% (14.5% to 25.3%) |  | 24/207 (11.6%) | 12.2% (7.7% to 16.8%) |  |
| Alcoholic drinks | Never | 62/373 (16.6%) | 16.6% (13.0% to 20.2%) | 0.624 | 31/373 (8.3%) | 8.3% (5.6% to 11.0%) | - |
|  | 1-3 times a month | 1/6 (16.7%) | 30.6% (0.0% to 68.8%) |  | 0/6 (0.0%) | excluded |  |
|  | Few times a day | 1/2 (50.0%) | 27.4% (0.0% to 77.0%) |  | 0/2 (0.0%) | excluded |  |
| satisfaction with own figure | no | 43/145 (29.7%) | 30.0% (22.9% to 37.1%) | **<0.001** | 23/145 (15.9%) | 17.3% (11.5% to 23.0%) | **<0.001** |
|  | yes | 21/236 (8.9%) | 8.8% (5.2% to 12.4%) |  | 8/236 (3.4%) | 3.2% (1.1% to 5.3%) |  |
| a strong fear of gaining weight | no | 37/273 (13.6%) | 13.6% (9.7% to 17.5%) | **0.013** | 17/273 (6.2%) | 6.0% (3.3% to 8.7%) | **0.019** |
|  | yes | 27/108 (25.0%) | 24.8% (16.6% to 33.0%) |  | 14/108 (13.0%) | 13.7% (7.2% to 20.2%) |  |
| exercising after eating to avoid gaining weight | no | 40/290 (13.8%) | 14.4% (10.5% to 18.4%) | **0.043** | 14/290 (4.8%) | 5.1% (2.5% to 7.7%) | **0.002** |
|  | yes | 24/91 (26.4%) | 23.8% (14.9% to 32.6%) |  | 17/91 (18.7%) | 16.2% (8.5% to 23.9%) |  |
| Any weight loss diet | no | 37/325 (11.4%) | 11.5% (8.1% to 14.9%) | **<0.001** | 14/325 (4.3%) | 4.2% (2.1% to 6.2%) | **<0.001** |
|  | yes | 27/56 (48.2%) | 46.8% (34.8% to 58.9%) |  | 17/56 (30.4%) | 31.5% (19.7% to 43.3%) |  |
| encountering comments about own appearance | no | 29/249 (11.6%) | 11.6% (7.8% to 15.5%) | **0.001** | 14/249 (5.6%) | 5.4% (2.7% to 8.0%) | **0.004** |
|  | yes | 35/132 (26.5%) | 26.9% (19.7% to 34.0%) |  | 17/132 (12.9%) | 14.1% (8.4% to 19.9%) |  |
| Accepting own figure but, starting weight loss diet due to the pressure of the environment, mass media, etc. | no | 57/365 (15.6%) | 15.6% (12.1% to 19.2%) | **0.002** | 25/365 (6.8%) | 6.8% (4.4% to 9.3%) | **<0.001** |
|  | yes | 7/16 (43.8%) | 44.2% (23.1% to 65.4%) |  | 6/16 (37.5%) | 34.9% (18.7% to 51.2%) |  |
| A slim figure indicates good health | completely disagree | 3/27 (11.1%) | 10.9% (0.3% to 21.5%) | 0.375 | 3/27 (11.1%) | 9.6% (0.8% to 18.4%) | 0.575 |
|  | rather disagree | 14/70 (20.0%) | 22.8% (13.2% to 32.5%) |  | 3/70 (4.3%) | 5.5% (0.0% to 11.3%) |  |
|  | no opinion | 13/80 (16.3%) | 15.8% (7.9% to 23.8%) |  | 5/80 (6.3%) | 4.9% (0.3% to 9.4%) |  |
|  | rather agree | 21/146 (14.4%) | 14.2% (8.9% to 19.4%) |  | 14/146 (9.6%) | 10.1% (5.6% to 14.5%) |  |
|  | completely agree | 13/58 (22.4%) | 21.1% (10.8% to 31.3%) |  | 6/58 (10.3%) | 10.4% (2.6% to 18.2%) |  |
| An obese figure is unsightly, but it is not harmful to health | completely disagree | 16/91 (17.6%) | 20.0% (11.1% to 28.9%) | 0.739 | 9/91 (9.9%) | 11.7% (4.6% to 18.8%) | 0.658 |
|  | rather disagree | 19/125 (15.2%) | 13.8% (8.2% to 19.4%) |  | 10/125 (8.0%) | 8.3% (3.6% to 13.1%) |  |
|  | no opinion | 14/73 (19.2%) | 18.0% (10.1% to 26.0%) |  | 6/73 (8.2%) | 5.9% (1.6% to 10.3%) |  |
|  | rather agree | 10/60 (16.7%) | 19.4% (9.8% to 29.0%) |  | 4/60 (6.7%) | 8.8% (1.6% to 16.0%) |  |
|  | completely agree | 5/32 (15.6%) | 14.5% (2.6% to 26.3%) |  | 2/32 (6.3%) | 5.0% (0.0% to 12.8%) |  |
| obese people have fewer friends | completely disagree | 30/146 (20.5%) | 20.0% (13.7% to 26.2%) | 0.263 | 16/146 (11.0%) | 10.6% (5.9% to 15.3%) | 0.272 |
|  | rather disagree | 15/85 (17.6%) | 18.7% (10.3% to 27.0%) |  | 4/85 (4.7%) | 5.7% (0.6% to 10.9%) |  |
|  | no opinion | 9/106 (8.5%) | 9.4% (3.4% to 15.5%) |  | 4/106 (3.8%) | 4.0% (0.1% to 7.8%) |  |
|  | rather agree | 5/31 (16.1%) | 15.4% (3.2% to 27.6%) |  | 4/31 (12.9%) | 12.4% (0.9% to 23.9%) |  |
|  | completely agree | 5/13 (38.5%) | 26.1% (3.8% to 48.3%) |  | 3/13 (23.1%) | 13.2% (0.0% to 30.5%) |  |
| Excessively skinny figure is unsightly | completely disagree | 7/48 (14.6%) | 15.6% (5.9% to 25.3%) | 0.835 | 2/48 (4.2%) | 4.3% (0.0% to 9.7%) | 0.379 |
|  | rather disagree | 6/47 (12.8%) | 12.7% (2.3% to 23.2%) |  | 4/47 (8.5%) | 7.8% (0.0% to 16.0%) |  |
|  | no opinion | 15/93 (16.1%) | 16.7% (9.3% to 24.2%) |  | 9/93 (9.7%) | 8.8% (3.1% to 14.6%) |  |
|  | rather agree | 14/89 (15.7%) | 15.7% (8.6% to 22.9%) |  | 4/89 (4.5%) | 5.0% (0.0% to 10.0%) |  |
|  | completely agree | 22/104 (21.2%) | 20.2% (12.6% to 27.8%) |  | 12/104 (11.5%) | 12.1% (5.8% to 18.4%) |  |
| A very thin figure indicates malnutrition | completely disagree | 6/46 (13.0%) | 15.2% (5.8% to 24.7%) | 0.136 | 3/46 (6.5%) | 8.4% (0.9% to 15.8%) | 0.592 |
|  | rather disagree | 12/104 (11.5%) | 12.5% (5.7% to 19.3%) |  | 6/104 (5.8%) | 6.3% (0.5% to 12.0%) |  |
|  | no opinion | 25/115 (21.7%) | 21.0% (14.0% to 28.0%) |  | 13/115 (11.3%) | 10.5% (5.5% to 15.5%) |  |
|  | rather agree | 13/89 (14.6%) | 13.1% (6.8% to 19.4%) |  | 6/89 (6.7%) | 5.8% (1.5% to 10.2%) |  |
|  | completely agree | 8/27 (29.6%) | 30.0% (13.0% to 47.1%) |  | 3/27 (11.1%) | 12.9% (1.0% to 24.8%) |  |
| Obesity is the cause of disease | completely disagree | 6/41 (14.6%) | 16.5% (5.3% to 27.7%) | 0.895 | 4/41 (9.8%) | 11.8% (2.9% to 20.8%) | 0.307 |
|  | rather disagree | 10/56 (17.9%) | 18.2% (6.8% to 29.6%) |  | 4/56 (7.1%) | 6.5% (0.0% to 13.1%) |  |
|  | no opinion | 17/95 (17.9%) | 17.9% (10.9% to 24.8%) |  | 9/95 (9.5%) | 8.8% (3.8% to 13.7%) |  |
|  | rather agree | 18/121 (14.9%) | 14.2% (8.3% to 20.2%) |  | 6/121 (5.0%) | 4.6% (0.7% to 8.5%) |  |
|  | completely agree | 13/68 (19.1%) | 19.1% (10.2% to 28.0%) |  | 8/68 (11.8%) | 13.5% (5.6% to 21.5%) |  |
| Thinness is trendy | completely disagree | 14/53 (26.4%) | 29.9% (18.1% to 41.7%) | **0.037** | 6/53 (11.3%) | 16.1% (6.2% to 26.0%) | 0.087 |
|  | rather disagree | 5/60 (8.3%) | 9.0% (1.3% to 16.7%) |  | 3/60 (5.0%) | 6.6% (0.0% to 13.5%) |  |
|  | no opinion | 25/165 (15.2%) | 14.6% (9.5% to 19.6%) |  | 11/165 (6.7%) | 5.4% (2.1% to 8.6%) |  |
|  | rather agree | 11/74 (14.9%) | 15.1% (7.2% to 22.9%) |  | 5/74 (6.8%) | 7.6% (1.4% to 13.7%) |  |
|  | completely agree | 9/29 (31.0%) | 25.9% (12.6% to 39.2%) |  | 6/29 (20.7%) | 16.7% (6.8% to 26.5%) |  |
| Being overweight means you don't care about your appearance | completely disagree | 13/51 (25.5%) | 24.7% (14.6% to 34.8%) | 0.294 | 9/51 (17.6%) | 17.5% (8.9% to 26.2%) | 0.057 |
|  | rather disagree | 15/101 (14.9%) | 14.5% (7.7% to 21.3%) |  | 4/101 (4.0%) | 4.2% (0.1% to 8.2%) |  |
|  | no opinion | 22/128 (17.2%) | 17.9% (11.1% to 24.7%) |  | 9/128 (7.0%) | 6.5% (2.6% to 10.4%) |  |
|  | rather agree | 10/81 (12.3%) | 12.0% (5.2% to 18.9%) |  | 7/81 (8.6%) | 9.1% (2.7% to 15.6%) |  |
|  | completely agree | 4/20 (20.0%) | 21.2% (3.1% to 39.3%) |  | 2/20 (10.0%) | 10.5% (0.0% to 23.2%) |  |
| A slim figure guarantees success in life | completely disagree | 15/51 (29.4%) | 27.6% (16.0% to 39.2%) | **0.032** | 7/51 (13.7%) | 12.8% (4.3% to 21.4%) | 0.543 |
|  | rather disagree | 17/91 (18.7%) | 18.8% (10.5% to 27.0%) |  | 6/91 (6.6%) | 6.3% (1.2% to 11.4%) |  |
|  | no opinion | 13/135 (9.6%) | 10.0% (5.1% to 14.8%) |  | 9/135 (6.7%) | 6.7% (2.6% to 10.8%) |  |
|  | rather agree | 13/77 (16.9%) | 16.3% (8.3% to 24.3%) |  | 6/77 (7.8%) | 8.3% (1.7% to 14.9%) |  |
|  | completely agree | 6/27 (22.2%) | 23.8% (7.9% to 39.6%) |  | 3/27 (11.1%) | 12.2% (0.6% to 23.7%) |  |
| Perception of own figure | underweight | 0/34 (0.0%) | excluded | **<0.001** | 0/34 (0.0%) | excluded | **<0.001** |
|  | risk of underweight | 3/136 (2.2%) | 2.4% (0.0% to 5.1%) |  | 2/136 (1.5%) | 1.6% (0.0% to 3.5%) |  |
|  | standard | 24/144 (16.7%) | 16.3% (10.6% to 21.9%) |  | 8/144 (5.6%) | 5.4% (1.4% to 9.4%) |  |
|  | overweight | 29/57 (50.9%) | 49.9% (37.3% to 62.4%) |  | 15/57 (26.3%) | 25.2% (15.8% to 34.7%) |  |
|  | obesity | 8/10 (80.0%) | 78.9% (54.6% to 103.3%) |  | 6/10 (60.0%) | 71.6% (48.9% to 94.2%) |  |
| Ideal figure | underweight | 4/43 (9.3%) | 12.0% (2.4% to 21.6%) | **0.002** | 2/43 (4.7%) | 6.6% (0.0% to 14.2%) | **0.002** |
|  | risk of underweight | 33/192 (17.2%) | 16.4% (11.4% to 21.3%) |  | 15/192 (7.8%) | 7.3% (3.7% to 10.8%) |  |
|  | standard | 23/139 (16.5%) | 16.4% (10.6% to 22.2%) |  | 12/139 (8.6%) | 8.5% (4.1% to 13.0%) |  |
|  | overweight | 4/6 (66.7%) | 73.9% (47.1% to 100.7%) |  | 2/6 (33.3%) | 50.6% (23.6% to 77.6%) |  |
|  | obesity | 0/1 (0.0%) | excluded |  | 0/1 (0.0%) | excluded |  |
| Any diet | no | 32/297 (10.8%) | 11.0% (7.5% to 14.5%) | **<0.001** | 12/297 (4.0%) | 3.9% (1.8% to 5.9%) | **<0.001** |
|  | yes, health reason | 8/26 (30.8%) | 31.0% (13.9% to 48.0%) |  | 5/26 (19.2%) | 22.1% (7.8% to 36.3%) |  |
|  | yes, own decision | 24/58 (41.4%) | 38.6% (27.0% to 50.2%) |  | 14/58 (24.1%) | 23.9% (13.7% to 34.1%) |  |
| eating meals outside the home | Several times a day | 3/29 (10.3%) | 9.7% (0.0% to 21.2%) | 0.450 | 1/29 (3.4%) | 2.3% (0.0% to 7.5%) | 0.069 |
|  | Once a day | 8/60 (13.3%) | 14.5% (5.7% to 23.4%) |  | 5/60 (8.3%) | 10.9% (2.6% to 19.2%) |  |
|  | Several times a week | 9/70 (12.9%) | 13.0% (5.1% to 20.9%) |  | 4/70 (5.7%) | 5.6% (0.5% to 10.8%) |  |
|  | Once a week | 11/78 (14.1%) | 15.8% (7.8% to 23.8%) |  | 2/78 (2.6%) | 2.8% (0.0% to 6.5%) |  |
|  | 1-3 times a month | 28/117 (23.9%) | 22.2% (15.3% to 29.1%) |  | 14/117 (12.0%) | 11.2% (5.8% to 16.7%) |  |
|  | Never | 5/27 (18.5%) | 18.0% (5.4% to 30.6%) |  | 5/27 (18.5%) | 19.2% (7.2% to 31.2%) |  |
| ever tried smoking cigarettes | No | 59/351 (16.8%) | 16.8% (13.1% to 20.5%) | 0.944 | 29/351 (8.3%) | 8.3% (5.5% to 11.1%) | 0.750 |
|  | Yes | 5/30 (16.7%) | 17.3% (2.9% to 31.8%) |  | 2/30 (6.7%) | 6.7% (-2.0% to 15.4%) |  |
| ever tried drinking alcohol | No | 55/340 (16.2%) | 16.1% (12.5% to 19.8%) | 0.300 | 28/340 (8.2%) | 8.1% (5.3% to 10.9%) | 0.894 |
|  | Yes | 9/41 (22.0%) | 23.5% (8.6% to 38.5%) |  | 3/41 (7.3%) | 8.8% (-1.5% to 19.2%) |  |
| hours sleeping a day on weekdays | 6 or less hours/day | 11/63 (17.5%) | 18.0% (9.3% to 26.7%) | 0.581 | 7/63 (11.1%) | 13.1% (5.6% to 20.5%) | 0.141 |
|  | more than 6, but less than 9 hours/day | 45/270 (16.7%) | 17.6% (13.1% to 22.1%) |  | 20/270 (7.4%) | 8.2% (4.9% to 11.6%) |  |
|  | 9 or more hours/day | 8/48 (16.7%) | 12.0% (3.6% to 20.4%) |  | 4/48 (8.3%) | 4.2% (0.0% to 8.3%) |  |
| hours sleeping a day on weekend | 6 or less hours/day | 5/45 (11.1%) | 11.3% (2.6% to 19.9%) | 0.460 | 3/45 (6.7%) | 6.2% (-1.7% to 14.2%) | 0.684 |
|  | more than 6, but less than 9 hours/day | 24/156 (15.4%) | 16.2% (10.6% to 21.9%) |  | 10/156 (6.4%) | 7.2% (3.2% to 11.2%) |  |
|  | 9 or more hours/day | 35/180 (19.4%) | 18.6% (13.0% to 24.2%) |  | 18/180 (10.0%) | 9.3% (5.4% to 13.2%) |  |
| hours a day in front of the TV or computer | from 8 to almost 10 hours | 1/3 (33.3%) | 34.6% (0.0% to 91.0%) | 0.509 | 0/3 (0.0%) | excluded | 0.129 |
|  | from 6 to almost 8 hours | 1/16 (6.3%) | 5.5% (0.0% to 14.5%) |  | 0/16 (0.0%) | excluded |  |
|  | from 4 to almost 6 hours | 7/52 (13.5%) | 14.8% (5.3% to 24.2%) |  | 2/52 (3.8%) | 3.7% (0.0% to 8.0%) |  |
|  | from 2 to almost 4 hours | 28/158 (17.7%) | 16.6% (11.1% to 22.0%) |  | 13/158 (8.2%) | 7.6% (3.6% to 11.6%) |  |
|  | Less than 2 hours | 27/152 (17.8%) | 18.8% (12.5% to 25.1%) |  | 16/152 (10.5%) | 11.4% (6.7% to 16.1%) |  |
| physical activity at school | low | 14/59 (23.7%) | 26.5% (15.1% to 37.8%) | **0.025** | 5/59 (8.5%) | 10.0% (2.0% to 18.0%) | 0.550 |
|  | moderate | 34/167 (20.4%) | 18.5% (13.0% to 24.1%) |  | 17/167 (10.2%) | 9.1% (4.9% to 13.3%) |  |
|  | high | 16/155 (10.3%) | 11.2% (6.2% to 16.2%) |  | 9/155 (5.8%) | 6.4% (2.5% to 10.2%) |  |
| physical activity at home | low | 14/70 (20.0%) | 21.1% (12.0% to 30.2%) | 0.485 | 6/70 (8.6%) | 8.1% (2.2% to 13.9%) | 0.494 |
|  | moderate | 36/208 (17.3%) | 16.7% (11.9% to 21.5%) |  | 19/208 (9.1%) | 9.5% (5.7% to 13.4%) |  |
|  | high | 14/103 (13.6%) | 14.2% (6.9% to 21.4%) |  | 6/103 (5.8%) | 5.6% (1.0% to 10.1%) |  |
| pHDI-10 category | low | 46/317 (14.5%) | 14.8% (11.1% to 18.6%) | 0.111 | 24/317 (7.6%) | 7.7% (4.9% to 10.5%) | 0.440 |
|  | moderate | 17/60 (28.3%) | 26.2% (14.9% to 37.5%) |  | 7/60 (11.7%) | 10.9% (2.2% to 19.7%) |  |
|  | high | 1/4 (25.0%) | 20.1% (0.0% to 49.8%) |  | 0/4 (0.0%) | excluded |  |
| nHDI-11 category | low | 1/2 (50.0%) | 33.6% (0.0% to 83.1%) | 0.727 | 0/2 (0.0%) | excluded | 0.901 |
|  | moderate | 4/27 (14.8%) | 16.8% (1.1% to 32.5%) |  | 2/27 (7.4%) | 9.0% (0.0% to 22.0%) |  |
|  | high | 59/352 (16.8%) | 16.7% (13.0% to 20.4%) |  | 29/352 (8.2%) | 8.1% (5.4% to 10.9%) |  |

^a^ adjusted for potential difference in age, sex, SBP, DBP, place of living, number of siblings, parents work status, and self-reported financial situation between subcategories (predictive margins from logistic regression models)

^b^ overall variable effect in logistic regression model
